# Data to understand the nature of non-covalent interactions in the thiophene clusters

**DOI:** 10.1016/j.dib.2022.107818

**Published:** 2022-01-10

**Authors:** Alhadji Malloum, Jeanet Conradie

**Affiliations:** a Department of Chemistry, University of the Free State, PO BOX 339, Bloemfontein 9300, South Africa; b Department of Physics, Faculty of Science, University of Maroua, PO BOX 46, Maroua, Cameroon; c Department of Chemistry, UiT - The Arctic University of Norway, Tromsø N-9037, Norway

**Keywords:** Thiophene clusters, Non-covalent interactions, QTAIM analysis, Heterocyclic molecules, Benzene clusters

## Abstract

We have reported herein the data to understand the nature and number of non-covalent interactions that stabilize the structures of the thiophene clusters. In addition, we have also provided the optimized Cartesian coordinates of all the structures of the investigated thiophene clusters. Initially, the geometries have been generated using the ABCluster code which performs a global optimization to locate local and global minima structures of molecular clusters. The located geometries have been optimized at the MP2/aug-cc-pVDZ level of theory using Gaussian 16 suite of programs. To understand the nature of non-covalent interactions, we have performed a quantum theory of atoms in molecules (QTAIM) analysis on all the structures of the thiophene dimer. Furthermore, the QTAIM analysis has been performed also on the most stable structure of the thiophene trimer and tetramer. We have used the AIMAll program to perform the QTAIM analysis. The data reported in this paper contains the critical points, the bonds paths and their related properties, for each investigated structures. Besides, the data contains the optimized Cartesian coordinates of all the investigated structures of the thiophene clusters. This can be use for any further investigations involving thiophene clusters. For further information and analysis, the reader is referred to the original related research article (Malloum and Conradie, 2022).

**Specifications Table**

**Table:** 

Subject	Chemistry
Specific subject area	Physical and Theoretical Chemistry
Type of data	Table Figure
How data were acquired	Data were obtain using Gaussian 16 computational chemistry program. Data for quantum theory of atoms in molecule analysis (QTAIM) are calculated using AIMAll program.
Data format	Raw Analyzed
Parameters for data collection	Raw data (optimized Cartesian coordinates of the structures) are extracted directly from the Gaussian output files. Analyzed data (description of critical points and their related figures) were obtained from the AIMAll program.
Description of data collection	Optimization of the geometries have been performed using the resources of the Center of High Performance Computing (CHPC), South Africa. The QTAIM analysis has been performed in our laboratory (Physical Chemistry Laboratory of the Department of Chemistry).
Data source location	Institution: Department of Chemistry, University of the Free State City/Town/Region: Bloemfontein Country: South Africa
Data accessibility	With the article
Related research article	Alhadji Malloum and Jeanet Conradie, Non-Covalent Interactions in Small Thiophene Clusters, J. Mol. Liq. 347 (2022) 118301 [1]. https://doi.org/10.1016/j.molliq.2021.118301

## Value of the Data

• We have reported the critical points, bond paths and their related properties for the structures of the thiophene dimer. These data will be useful to understand the nature and number of non-covalent interactions that stabilize the structures of the thiophene clusters.
• The optimized Cartesian coordinates of the thiophene clusters will save computational time for all further investigations involving thiophene clusters.
• Using this data one would be able to determine all non-covalent bonds in thiophene clusters and therefore, will provide proper understanding of non-covalent bonds in aromatic and heterocyclic molecular clusters.
• The optimized Cartesian coordinates of the structures of the thiophene clusters will be useful for investigations involving solvation processes and/or ions transfer in thiophene solvent.
• The data provided in this paper will be of great interest for investigations using quantum cluster equilibrium theory to compute the thermodynamics properties of the liquid thiophene.

## 1 Data Description

**Fig. 1:**
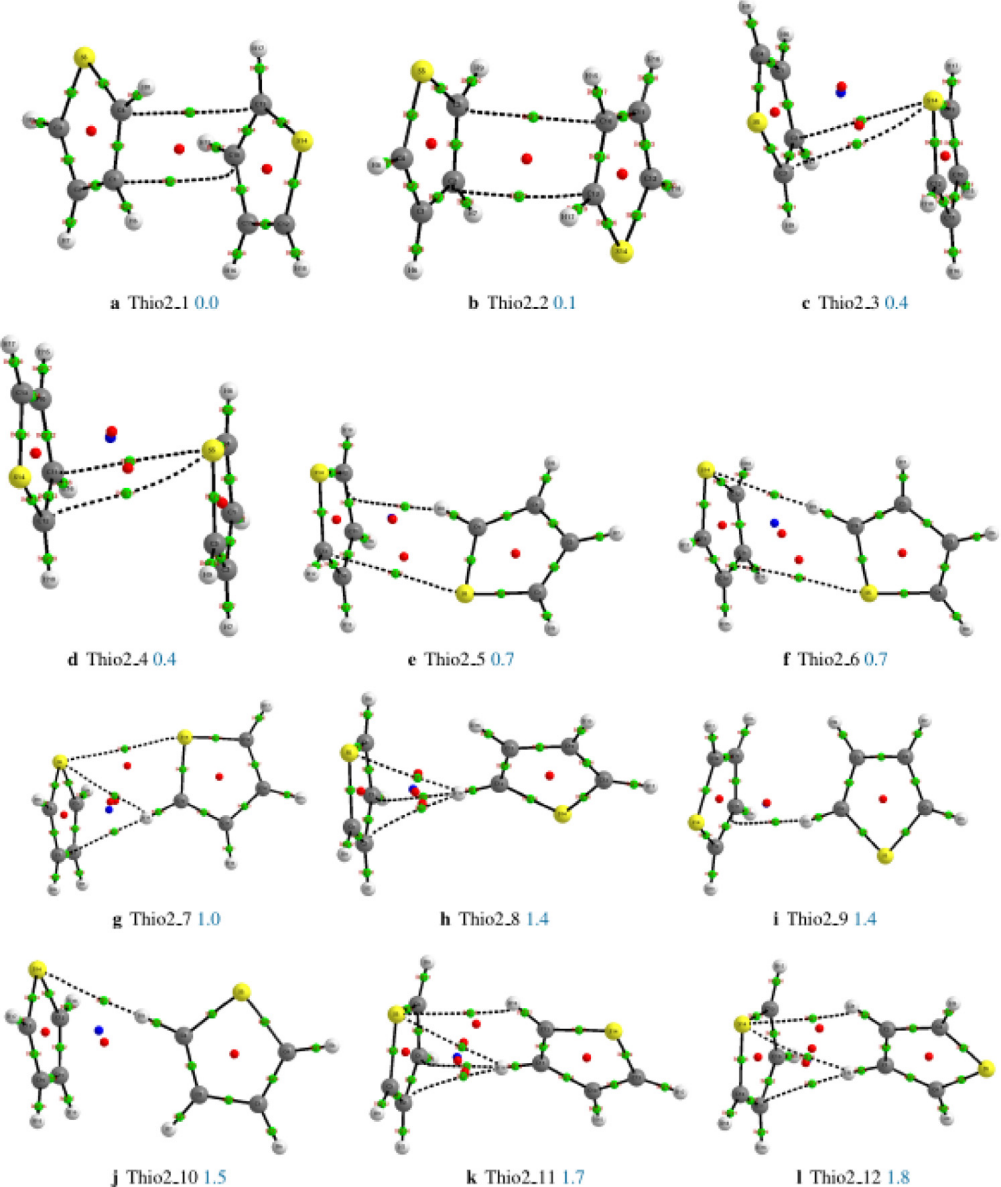
Critical points and bond paths of all the investigated structures of the thiophene dimer. Bond critical points (BCP) are reported in green color, Ring critical points (RCP) are in red color while cage critical points (CCPs) are in blue color. Structures of **Thio2_1** and **Thio2_10** are also reported in [1].

We have reported in Fig. 1 the structures of the thiophene dimer, their relative energies, critical points, and bond paths. Each sub-caption of the structure reports the name and the relative energy in kcal/mol as calculated at the MP2/aug-cc-pVDZ level of theory. For each structure, critical points (Bond critical points, BCP, are reported in green color, Ring critical points, RCP, are in red color while cage critical points, CCPs are in blue color) and bond paths (covalent bonds in solid lines and non-covalent bonds in dash lines) are also given in the figure. The properties of the critical points of all the structures of the thiophene dimer, thiophene trimer, and those of the most stable structures of the thiophene tetramer are reported in Excel files as supplementary material. Each of the tables in the Excel file has seven columns which describe respectively, the name of the bond critical point (**name**), the two atoms involved in the bond critical point (**Atoms**), the electron density at the bond critical point (ρ), the Laplacian of the electron density at the bond critical point ($\nabla^{2}\rho$), the ellipticity of the bond (**Ellipticity**), the electron kinetic energy density at the bond critical point (**K**) and the difference between the bond path length with a corresponding geometric bond length (**BPL-GBL_I**). In the supplementary material, we have also reported the Cartesian coordinates of all the investigated structures of the thiophene dimer, trimer and tetramer as optimized at the MP2/aug-cc-pVDZ level of theory.

## 2 Experimental Design, Materials and Methods

The most important task in exploring the potential energy surfaces (PESs) of molecular clusters is the location of possible stable structures. Thus, to obtain the Cartesian coordinates of the structures reported in the supplementary material, we explored thoroughly the PESs of thiophene clusters from dimer to tetramer using classical molecular dynamics as implemented in ABCluster [2], [3]. ABCluster generates possible structures and classifies them following their relative energies based on classical potential energy (U). The classical potential energy (U) implemented in ABCluster is constituted of Lenard-Jones and electrostatics interactions:

(1) $$U=\sum_{I=1}^{N}\sum_{J<I}^{N}\sum_{i_{I}}\sum_{j_{J}}\left(\frac{e^{2}}{4\pi \epsilon_{0}}\frac{q_{i_{I}}q_{j_{J}}}{r_{i_{I}j_{J}}}+4\epsilon_{i_{I}j_{J}}\left({\left(\frac{\sigma_{i_{I}j_{J}}}{r_{i_{I}j_{J}}}\right)}^{12}-{\left(\frac{\sigma_{i_{I}j_{J}}}{r_{i_{I}j_{J}}}\right)}^{6}\right)\right).$$

Where I and J are the indices of the molecules, $i_{I}$ and $j_{J}$ are the indices of the atoms in molecules I and J, respectively. $r_{i_{I}j_{J}}$ is the distance between atom $i_{I}$ and $j_{J}$. The accuracy of the exploration of a given PES using ABCluster is based on the setting of three parameters: the scout limit $g_{limit}$, the size of the population of trial solutions SN and the maximum cycle number $g_{\max}$. For the potential energy surfaces of thiophene clusters, we used $g_{limit}=4$, SN=60 and $g_{\max}=5000$. For each cluster size n, we generated 500 geometries using ABCluster. ABCluster is based on the bee colony algorithm. The authors of ABCluster have stated that a scout limit $g_{limit}$ between 3 and 5 is enough to achieve accurate exploration of a given PES. It is worth noting that ABCluster has been used in our previous works to explore PESs of molecular clusters [4], [5], [6], [7], [8]. For further reading on ABCluster, the reader is referred to our previous works and the original works of Zhang and Dolg [2], [3]. In practical use, ABCluster generates several geometries that are similar one to another. Thus, from the generated geometries, we selected those that are different one from another. The selected geometries are fully optimized at the MP2/aug-cc-pVDZ level of theory. Optimizations have been performed using Gaussian 16 suite of program. We used the **tight** option of the optimization for high accuracy. To ensure accurate location of stable minima, frequencies calculations have been also performed at the same level of theory. All the located structures have positive frequencies highlighting their stability. After optimizations, some structures have been optimized to the same isomer. The redundant structures have been eliminated from the final structures reported in the supplementary material.

After locating the structures, we used their Gaussian formatted checkpoint files to perform (to generate the data related to) the quantum theory of atoms in molecule (QTAIM) analysis. The QTAIM analysis is performed to provide data necessary to understand the nature of non-covalent bondings in thiophene clusters. QTAIM analyses the topology of the electron density, ρ, of a given molecule to locate its critical points. These critical points are obtained by vanishing the first order derivatives of the electron density ρ. Depending on the sign of the second order derivatives, there are usually four critical points used in QTAIM analysis: (3, -3) atom critical point (ACP); (3, -1) bond critical point (BCP); (3, 1) ring critical point (RCP); and (3, 3) cage critical point (CCP). A bond path connects two atom critical points along which lies a bond critical points. Bader [9] stated that the analysis of the topology of the electron density and the properties of bond critical points can provide universal description of chemical bondings. When the second order derivatives of the electron density (the Laplacian of the electron density $\nabla^{2}\rho$) is positive (respectively negative), it indicates that the electron is locally depleted (respectively concentrated) [10]. The values of ρ and $\nabla^{2}\rho$ at the BCP can be used to distinguish between different types of bonding (covalent, ionic bonding, hydrogen bonding, and van der Waals interactions) [11], [12]. The range proposed for ρ and $\nabla^{2}\rho$ at a bond critical point for a hydrogen bond to exist, is 0.002-0.035 $ea_{0}^{-3}$ for ρ and 0.024-0.139 $ea_{0}^{-5}$ for $\nabla^{2}\rho$ [13]. A positive value of $\nabla^{2}\rho$ at a bond critical point indicates a non-covalent bonding, while a negative value of $\nabla^{2}\rho$ at a bond critical point indicates a covalent bonding [14]. Generally, the larger the value of the electron density at a bond critical point, the stronger the corresponding bonding [15], [16]. Using the description above, one can now analyse the data provided as excel file in the supplementary material to understand the non-covalent interactions in thiophene clusters.

## CRediT authorship contribution statement

**Alhadji Malloum:** Conceptualization, Methodology, Validation, Formal analysis, Investigation, Data curation, Writing – original draft, Visualization. **Jeanet Conradie:** Resources, Visualization, Writing – review & editing, Supervision, Funding acquisition, Project administration.

## Declaration of Competing Interest

The authors declare that they have no known competing financial interests or personal relationships which have, or could be perceived to have, influenced the work reported in this article.

## Appendix A Supplementary materials

Supplementary Data S1 Supplementary Raw Research Data. This is open data under the CC BY license http://creativecommons.org/licenses/by/4.0/ Supplementary Data S1: Cartesian coordinates of the optimized structures of the thiophene clusters.

Supplementary Data S2 Supplementary Raw Research Data. This is open data under the CC BY license http://creativecommons.org/licenses/by/4.0/ Supplementary Data S2: QTAIM analysis data of the investigated structures of the thiophene clusters.

## References

[1] Malloum A., Conradie J. (2022). Non-covalent interactions in small thiophene clusters. J. Mol. Liq..

[2] Zhang J., Dolg M. (2015). Abcluster: the artificial bee colony algorithm for cluster global optimization. Phys. Chem. Chem. Phys..

[3] Zhang J., Dolg M. (2016). Global optimization of clusters of rigid molecules using the artificial bee colony algorithm. Phys. Chem. Chem. Phys..

[4] Malloum A., Fifen J.J., Dhaouadi Z., Engo S.G.N., Conradie J. (2019). Structures, relative stabilities and binding energies of neutral water clusters,(H${}_{2}$O)${}_{2-30}$. New J. Chem..

[5] Malloum A., Fifen J.J., Conradie J. (2020). Exploration of the potential energy surfaces of small ethanol clusters. Phys. Chem. Chem. Phys..

[6] Malloum A., Conradie J. (2020). Solvent effects on the structures of the neutral ammonia clusters. Comput. Theor. Chem..

[7] Malloum A., Conradie J. (2020). Global and local minima of protonated acetonitrile clusters. New J. Chem..

[8] Malloum A., Conradie J. (2021). Structures of water clusters in the solvent phase and relative stability compared to gas phase. Polyhedron.

[9] Bader R.F. (1998). A bond path: a universal indicator of bonded interactions. J. Phys. Chem. A.

[10] Bader R.F., Essén H. (1984). The characterization of atomic interactions. J. Chem. Phys..

[11] Grabowski S.J. (2011). What is the covalency of hydrogen bonding?. Chem. Rev..

[12] Parthasarathi R., Subramanian V., Sathyamurthy N. (2006). Hydrogen bonding without borders: an atoms-in-molecules perspective. J. Phys. Chem. A.

[13] Koch U., Popelier P.L. (1995). Characterization of cho hydrogen bonds on the basis of the charge density. J. Phys. Chem..

[14] Platts J.A., Overgaard J., Jones C., Iversen B.B., Stasch A. (2011). First experimental characterization of a non-nuclear attractor in a dimeric magnesium (i) compound. J. Phys. Chem. A.

[15] Bone R.G., Bader R.F. (1996). Identifying and analyzing intermolecular bonding interactions in Van Der Waals molecules. J. Phys. Chem..

[16] Alkorta I., Blanco F., Elguero J., Dobado J.A., Ferrer S.M., Vidal I. (2009). Carbon⋯carbon weak interactions. J. Phys. Chem. A.

