# Supplementary material for: Data to understand the nature of non-covalent interactions in the thiophene clusters
Source: Data Brief. 2022 Jan 10;40:107818. doi: 10.1016/j.dib.2022.107818 (PMC8762079; doi:10.1016/j.dib.2022.107818)
Supplement: Supplementary Data S1 — Supplementary Raw Research Data. This is open data under the CC BY license http://creativecommons.org/licenses/by/4.0/ [file mmc1.pdf]

Cartesian coordinates of : Thio2_ 1

Atomic number (AN) and Cartesian coordinates

AN	X	Y	Z
C	0.966639	0.574805	1.334154
C	1.641845	1.452031	0.434527
C	2.391027	0.767203	-0.517879
C	1.213700	-0.765511	1.054602
S	2.271747	-0.946372	-0.303532
H	0.317325	0.900304	2.148727
H	1.583915	2.542097	0.472179
H	0.831825	-1.646143	1.570637
H	3.003708	1.174514	-1.322418
C	-0.966642	0.574808	-1.334155
C	-1.641845	1.452032	-0.434525
C	-2.391025	0.767202	0.517882
C	-1.213704	-0.765508	-1.054605
S	-2.271745	-0.946373	0.303532
H	-0.317328	0.900308	-2.148728
H	-1.583918	2.542098	-0.472177
H	-0.831831	-1.646140	-1.570644
H	-3.003700	1.174511	1.322426

Cartesian coordinates of : Thio2_ 2

Atomic number (AN) and Cartesian coordinates

AN	X	Y	Z
C	-1.436601	1.567526	0.230157
C	-0.912248	0.727989	1.257410
C	-1.345466	-0.588819	1.139435
C	-2.261965	0.877982	-0.653742
S	-2.396459	-0.792366	-0.220275
H	-1.221872	2.634151	0.132960
H	-0.239956	1.062403	2.049555
H	-2.796192	1.260229	-1.523910
H	-1.103123	-1.440624	1.774182
C	0.983626	-1.074484	-1.033732
C	1.682355	-1.566518	0.109481
C	2.413465	-0.573642	0.755391
C	1.194482	0.284921	-1.241310
S	2.247895	0.953378	-0.042251
H	0.349272	-1.680904	-1.682735
H	1.658525	-2.603353	0.452413
H	0.785216	0.921895	-2.024595
H	3.039273	-0.659736	1.644006

Cartesian coordinates of : Thio2_ 3

```
 ---------------------------------------------------
 Atomic number (AN) and Cartesian coordinates
  AN         X            Y            Z
 ---------------------------------------------------
  C     -1.636735      1.545870     -0.008698
  C     -0.949332      0.977201     -1.122579
  C     -1.166927     -0.392459     -1.237911
  C     -2.360973      0.600004      0.710985
  S     -2.208285     -0.977486      0.014907
  H     -1.603622      2.602451      0.266853
  H     -0.317554      1.535926     -1.814826
  H     -2.970802      0.745451      1.602897
  H     -0.772145     -1.079586     -1.985447
  C      2.461189      0.755012     -0.707790
  C      2.356368     -0.642210     -0.971003
  C      1.671875     -1.321677      0.035113
  C      1.853924      1.114993      0.493835
  S      1.167905     -0.252475      1.294732
  H      2.952827      1.475095     -1.365669
  H      2.758344     -1.138755     -1.857074
  H      1.777634      2.104300      0.945193
  H      1.445058     -2.385909      0.102140
 ---------------------------------------------------

Cartesian coordinates of :   Thio2_ 4

 ---------------------------------------------------
 Atomic number (AN) and Cartesian coordinates
  AN         X            Y            Z
 ---------------------------------------------------
  C      2.461184     -0.755024      0.707781
  C      2.356369      0.642195      0.971010
  C      1.671878      1.321677     -0.035099
  C      1.853918     -1.114989     -0.493848
  S      1.167905      0.252491     -1.294730
  H      2.952819     -1.475116      1.365652
  H      2.758344      1.138728      1.857088
  H      1.777621     -2.104291     -0.945214
  H      1.445069      2.385911     -0.102115
  C     -1.636735     -1.545870      0.008685
  C     -0.949333     -0.977211      1.122572
  C     -1.166928      0.392448      1.237916
  C     -2.360969     -0.599997     -0.710992
  S     -2.208282      0.977487     -0.014899
  H     -1.603622     -2.602449     -0.266876
  H     -0.317556     -1.535942      1.814816
  H     -2.970796     -0.745436     -1.602906
  H     -0.772145      1.079568      1.985459
 ---------------------------------------------------

Cartesian coordinates of :   Thio2_ 5

 ---------------------------------------------------
```

Atomic number (AN) and Cartesian coordinates

AN	X	Y	Z
C	-2.183374	1.497362	0.145855
C	-3.420606	0.814554	-0.057558
C	-3.253149	-0.562822	-0.170137
C	-1.093181	0.633469	0.183738
S	-1.587074	-1.011693	-0.028192
H	-2.085535	2.579297	0.260367
H	-4.396141	1.302144	-0.119948
H	-0.038948	0.866806	0.329008
H	-4.015300	-1.326351	-0.328834
C	1.773079	-1.092466	0.935359
C	2.209188	0.164075	1.451459
C	2.551778	1.063262	0.443603
C	1.791953	-1.130918	-0.457230
S	2.345976	0.364667	-1.126639
H	1.449526	-1.936086	1.548523
H	2.264963	0.412430	2.513351
H	1.509743	-1.951171	-1.117242
H	2.915140	2.086259	0.541528

Cartesian coordinates of : Thio2_ 6

Atomic number (AN) and Cartesian coordinates

AN	X	Y	Z
C	3.426222	0.890624	-0.000001
C	2.154068	1.538780	-0.000003
C	1.093407	0.638195	-0.000002
C	3.313836	-0.496956	0.000003
S	1.656608	-0.997762	0.000003
H	4.387644	1.409223	-0.000001
H	2.011220	2.621763	-0.000005
H	4.111247	-1.240683	0.000005
H	0.022946	0.838532	-0.000001
C	-1.693316	-1.251880	0.713777
C	-1.693320	-1.251864	-0.713807
C	-2.202635	-0.070548	-1.246206
C	-2.202627	-0.070575	1.246205
S	-2.682328	1.031990	0.000013
H	-1.331662	-2.074861	1.333627
H	-1.331667	-2.074831	-1.333677
H	-2.315998	0.209250	2.293498
H	-2.316009	0.209302	-2.293493

Cartesian coordinates of : Thio2_ 7

Atomic number (AN) and Cartesian coordinates

AN	X	Y	Z

```
 ---------------------------------------------------
  C     -2.429678      1.245754     -0.713733
  C     -2.429629      1.245652      0.713938
  C     -2.190680     -0.019336      1.245647
  C     -2.190766     -0.019158     -1.245639
  S     -1.972610     -1.200036     -0.000088
  H     -2.590467      2.129915     -1.333990
  H     -2.590373      2.129724      1.334332
  H     -2.126636     -0.314313     -2.292895
  H     -2.126476     -0.314640      2.292856
  C      3.429721      0.816382     -0.000015
  C      2.170353      1.488666     -0.000039
  C      1.092286      0.608617     -0.000028
  C      3.290881     -0.568877      0.000017
  S      1.624457     -1.038268      0.000020
  H      4.400899      1.316537     -0.000021
  H      2.048225      2.574194     -0.000067
  H      4.073922     -1.327688      0.000039
  H      0.026411      0.832926     -0.000040
 ---------------------------------------------------

Cartesian coordinates of :   Thio2_ 8

 ---------------------------------------------------
 Atomic number (AN) and Cartesian coordinates
  AN         X             Y             Z
 ---------------------------------------------------
  C      2.296761      1.288791     -0.713574
  C      2.296655      1.288572      0.714026
  C      2.291294      0.001292      1.245836
  C      2.291478      0.001673     -1.245780
  S      2.291495     -1.199234     -0.000155
  H      2.294128      2.187479     -1.333698
  H      2.293927      2.187071      1.334424
  H      2.281974     -0.299801     -2.293100
  H      2.281630     -0.300501      2.293062
  C     -2.964523     -1.310737      0.000075
  C     -1.538079     -1.262697     -0.000032
  C     -1.045977      0.038957     -0.000122
  C     -3.539027     -0.042412      0.000066
  S     -2.331195      1.197436     -0.000075
  H     -3.555241     -2.229577      0.000157
  H     -0.884693     -2.137858     -0.000046
  H     -4.596358      0.223628      0.000132
  H     -0.011665      0.377692     -0.000212
 ---------------------------------------------------

Cartesian coordinates of :   Thio2_ 9

 ---------------------------------------------------
 Atomic number (AN) and Cartesian coordinates
  AN         X             Y             Z
 ---------------------------------------------------
  C     -1.515212      1.234377      0.014201
```

```
  C     -2.921350      1.267570     -0.227103
  C     -3.498668      0.001420     -0.176944
  C     -1.040454     -0.053300      0.244817
  S     -2.318311     -1.217154      0.165154
  H     -0.863193      2.109987      0.022073
  H     -3.495996      2.174090     -0.430203
  H     -0.022567     -0.375713      0.458419
  H     -4.543364     -0.274411     -0.322507
  C      2.098274      1.247711      0.768350
  C      2.319067      0.006821      1.437285
  C      2.460768     -1.057765      0.549338
  C      2.075337      1.108152     -0.617250
  S      2.329243     -0.534323     -1.094970
  H      1.955985      2.205816      1.272609
  H      2.367083     -0.113201      2.521520
  H      1.921301      1.876504     -1.374964
  H      2.639280     -2.109350      0.773949
 ---------------------------------------------------

Cartesian coordinates of :   Thio2_10

 ---------------------------------------------------
 Atomic number (AN) and Cartesian coordinates
  AN         X            Y            Z
 ---------------------------------------------------
  C     -2.999817      1.239357      0.000007
  C     -1.572898      1.216706      0.000002
  C     -1.057214     -0.075563     -0.000005
  C     -3.550941     -0.039445      0.000004
  S     -2.320988     -1.257100     -0.000005
  H     -3.607115      2.147329      0.000013
  H     -0.935662      2.103315      0.000004
  H     -4.603161     -0.324923      0.000006
  H     -0.015255     -0.389582     -0.000010
  C      2.008754      1.310338      0.713550
  C      2.008758      1.310317     -0.713588
  C      2.302702      0.058022     -1.246118
  C      2.302694      0.058059      1.246118
  S      2.582458     -1.111359      0.000018
  H      1.797984      2.183425      1.334662
  H      1.797991      2.183386     -1.334727
  H      2.364734     -0.237140      2.293437
  H      2.364750     -0.237208     -2.293427
 ---------------------------------------------------

Cartesian coordinates of :   Thio2_11

 ---------------------------------------------------
 Atomic number (AN) and Cartesian coordinates
  AN         X            Y            Z
 ---------------------------------------------------
  C     -2.584379      1.196512      0.713822
  C     -2.584417      1.196563     -0.713703
  C     -2.373745     -0.072903     -1.245954
```

C	-2.373679	-0.072993	1.245969
S	-2.183882	-1.258358	-0.000040
H	-2.725644	2.083941	1.334089
H	-2.725714	2.084037	-1.333899
H	-2.317088	-0.369515	2.293210
H	-2.317214	-0.369350	-2.293219
C	1.973752	1.486934	-0.000031
C	0.960539	0.482473	-0.000039
C	1.492158	-0.803679	-0.000014
C	3.259392	0.952205	-0.000001
S	3.223146	-0.778214	0.000017
H	1.778385	2.561720	-0.000046
H	-0.112001	0.680110	-0.000061
H	4.214771	1.477514	0.000011
H	0.958538	-1.753969	-0.000014

Cartesian coordinates of : Thio2_12

Atomic number (AN) and Cartesian coordinates

AN	X	Y	Z
C	0.959377	0.254047	-0.078620
C	1.422606	-1.094212	-0.017803
C	2.810908	-1.182261	0.036432
C	2.005517	1.171723	-0.070305
S	3.545306	0.384922	0.012659
H	-0.090308	0.546081	-0.126193
H	0.768791	-1.968917	-0.014797
H	1.951837	2.260064	-0.107391
H	3.434607	-2.074973	0.088202
C	-2.452705	1.234728	0.822545
C	-2.528063	1.335233	-0.599249
C	-2.472176	0.091756	-1.224393
C	-2.341010	-0.083423	1.257545
S	-2.335629	-1.190918	-0.071417
H	-2.471314	2.085745	1.506418
H	-2.614351	2.273414	-1.151168
H	-2.258251	-0.456496	2.278244
H	-2.502555	-0.134533	-2.290102

Cartesian coordinates of : Thio3_ 1

Atomic number (AN) and Cartesian coordinates

AN	X	Y	Z
C	0.870819	1.878863	1.416145
C	0.621642	3.116765	0.750241
C	1.157760	3.144574	-0.536825

```
  C      1.587461      0.980402      0.629974
  S      1.962414      1.657741     -0.916455
  H      0.534243      1.639651      2.427109
  H      0.071890      3.955778      1.182610
  H      1.902098     -0.036210      0.866364
  H      1.126645      3.952926     -1.267463
  C     -3.168542     -0.917724     -0.913505
  C     -2.134686     -0.153319     -1.534066
  C     -1.574968      0.789293     -0.674726
  C     -3.384639     -0.539301      0.411381
  S     -2.322045      0.744535      0.883125
  H     -3.733369     -1.713586     -1.403848
  H     -1.800473     -0.284445     -2.565606
  H     -4.108066     -0.941523      1.120889
  H     -0.770028      1.498988     -0.863875
  C      0.779303     -2.330744      1.511151
  C      2.124261     -2.553151      1.086576
  C      2.279984     -2.383893     -0.289382
  C     -0.066496     -1.991211      0.457639
  S      0.783799     -1.954091     -1.046090
  H      0.435709     -2.407749      2.545041
  H      2.949392     -2.823057      1.749318
  H     -1.131868     -1.761993      0.478892
  H      3.185769     -2.493066     -0.886318
 ---------------------------------------------------

Cartesian coordinates of :   Thio3_ 2

 ---------------------------------------------------
 Atomic number (AN) and Cartesian coordinates
  AN         X             Y             Z
 ---------------------------------------------------
  C      1.869567      1.628468      1.449457
  C      1.684367      2.945732      0.930503
  C      1.540108      2.953480     -0.456554
  C      1.858591      0.653088      0.455758
  S      1.633406      1.352207     -1.109927
  H      1.996870      1.392037      2.508040
  H      1.653314      3.854344      1.535732
  H      1.963071     -0.426921      0.557449
  H      1.388746      3.807158     -1.117389
  C     -3.393284     -0.014177      0.930493
  C     -2.345097      0.804830      1.449462
  C     -1.494896      1.283019      0.455773
  C     -3.327858     -0.142968     -0.456565
  S     -1.987756      0.738466     -1.109921
  H     -4.164654     -0.495363      1.535712
  H     -2.203993      1.033274      2.508049
  H     -3.991488     -0.700876     -1.117408
  H     -0.611812      1.913492      0.557472
  C      0.475531     -2.433314      1.449458
  C      1.708916     -2.931551      0.930498
  C      1.787748     -2.810495     -0.456559
  C     -0.363692     -1.936124      0.455761
```

```
  S      0.354349     -2.090667     -1.109927
  H      0.207127     -2.425351      2.508041
  H      2.511329     -3.358959      1.535724
  H     -1.351249     -1.486604      0.557452
  H      2.602736     -3.106248     -1.117396
 ---------------------------------------------------
```

```
Cartesian coordinates of :   Thio3_ 3

 ---------------------------------------------------
 Atomic number (AN) and Cartesian coordinates
  AN          X            Y            Z
 ---------------------------------------------------
  C     -2.898378     -1.819316     -1.039179
  C     -2.233587     -0.686907     -1.599936
  C     -1.901467      0.269032     -0.643448
  C     -3.068937     -1.703243      0.339906
  S     -2.410966     -0.216434      0.935108
  H     -3.239948     -2.685870     -1.609494
  H     -2.000755     -0.568486     -2.660633
  H     -3.542950     -2.406913      1.024596
  H     -1.394376      1.224583     -0.775219
  C      1.303878     -2.125129      1.283336
  C      0.528327     -1.970844      0.096065
  C      1.284991     -1.496780     -0.971132
  C      2.638771     -1.764045      1.104658
  S      2.939645     -1.248746     -0.522257
  H      0.908660     -2.477881      2.238684
  H     -0.540211     -2.178550      0.016053
  H      3.453040     -1.783546      1.829189
  H      0.953606     -1.271089     -1.984511
  C     -0.486987      3.266861      0.645150
  C     -0.025702      2.174495      1.440349
  C      0.920644      1.392519      0.782399
  C      0.122079      3.303534     -0.608568
  S      1.253665      2.005465     -0.799694
  H     -1.233335      3.997212      0.965133
  H     -0.373622      1.954861      2.452010
  H     -0.027912      4.023294     -1.413447
  H      1.418494      0.486756      1.129541
 ---------------------------------------------------
```

```
Cartesian coordinates of :   Thio3_ 4

 ---------------------------------------------------
 Atomic number (AN) and Cartesian coordinates
  AN          X            Y            Z
 ---------------------------------------------------
  C     -0.862727      1.808998     -0.128786
  C     -1.448275      1.123057     -1.232986
  C     -2.756398      0.714572     -0.980854
  C     -1.735591      1.914481      0.950888
  S     -3.267642      1.178092      0.608847
  H      0.157147      2.197937     -0.115129
```

H	-0.931591	0.922780	-2.173747
H	-1.561882	2.379999	1.921324
H	-3.440470	0.174783	-1.636111
C	0.294027	-2.131869	1.464500
C	0.983658	-3.161909	0.756140
C	0.500251	-3.327360	-0.541177
C	-0.695471	-1.522172	0.697248
S	-0.789575	-2.221647	-0.881024
H	0.513505	-1.832903	2.491728
H	1.801057	-3.758659	1.166707
H	-1.352893	-0.694990	0.962799
H	0.828682	-4.037270	-1.300498
C	2.214530	0.978011	-1.522939
C	2.618489	2.234288	-0.978047
C	2.702036	2.205230	0.413631
C	1.988991	0.015056	-0.542631
S	2.280481	0.646972	1.039559
H	2.087932	0.777860	-2.589286
H	2.839840	3.126229	-1.568204
H	1.678757	-1.023143	-0.659004
H	2.986579	3.010408	1.091389

Cartesian coordinates of : Thio3_ 5

Atomic number (AN) and Cartesian coordinates

AN	X	Y	Z
C	1.725424	1.790433	1.359912
C	0.795285	1.799353	0.278876
C	1.374685	1.390736	-0.919397
C	2.997637	1.371010	0.972350
S	3.050819	1.000981	-0.719392
H	1.481515	2.069643	2.387384
H	-0.257305	2.074048	0.366419
H	3.896590	1.269421	1.580529
H	0.904185	1.292939	-1.897635
C	0.257151	-2.445573	1.523151
C	-0.583709	-3.370020	0.831976
C	-0.522506	-3.208834	-0.551753
C	0.940593	-1.592470	0.660439
S	0.563037	-1.932936	-0.991645
H	0.356960	-2.392814	2.609459
H	-1.211271	-4.121859	1.315334
H	1.624036	-0.779478	0.904448
H	-1.046976	-3.773199	-1.323033
C	-2.793440	2.082948	0.859656
C	-2.433957	0.799859	1.372641
C	-2.079846	-0.099542	0.370810
C	-2.710935	2.140428	-0.530889
S	-2.197668	0.625256	-1.194641
H	-3.097945	2.934771	1.471619
H	-2.423783	0.537120	2.432613

```
 H     -2.925077      2.982899     -1.188661
 H     -1.758224     -1.136301      0.465730
 ---------------------------------------------------

Cartesian coordinates of :   Thio3_ 6

 ---------------------------------------------------
 Atomic number (AN) and Cartesian coordinates
  AN          X            Y            Z
 ---------------------------------------------------
  C      1.030112     -1.651194      0.023134
  C      1.469520     -1.079288     -1.207912
  C      2.777679     -0.603963     -1.144001
  C      2.014146     -1.606607      1.006456
  S      3.470173     -0.867509      0.422831
  H      0.035396     -2.066503      0.194406
  H      0.853355     -1.008811     -2.106624
  H      1.961315     -1.962487      2.035552
  H      3.366446     -0.121684     -1.924595
  C     -2.183068     -1.174433      1.479888
  C     -2.422093     -2.467462      0.923707
  C     -2.468492     -2.445350     -0.469605
  C     -2.046032     -0.187987      0.507056
  S     -2.221573     -0.844820     -1.082965
  H     -2.103428     -0.964816      2.548716
  H     -2.553103     -3.381327      1.507250
  H     -1.851901      0.876537      0.635845
  H     -2.635309     -3.275853     -1.155689
  C     -0.964724      3.255497      0.939911
  C      0.066799      2.444407      1.503203
  C      0.758002      1.710894      0.542423
  C     -1.037652      3.131144     -0.447067
  S      0.154308      2.026960     -1.046437
  H     -1.631303      3.902211      1.514520
  H      0.294794      2.385219      2.569624
  H     -1.718250      3.630969     -1.136579
  H      1.570285      0.998504      0.679546
 ---------------------------------------------------

Cartesian coordinates of :   Thio3_ 7

 ---------------------------------------------------
 Atomic number (AN) and Cartesian coordinates
  AN          X            Y            Z
 ---------------------------------------------------
  C     -0.871879     -1.649733     -0.363624
  C     -1.396241     -1.875441      0.942893
  C     -2.729618     -1.486550      1.061689
  C     -1.815942     -1.094575     -1.223333
  S     -3.338086     -0.851738     -0.431602
  H      0.155023     -1.867685     -0.661040
  H     -0.822074     -2.298101      1.769915
  H     -1.698234     -0.817427     -2.271112
  H     -3.381380     -1.542887      1.933724
```

```
  C      0.738162      3.320217      0.879310
  C     -0.074917      2.358603      1.552351
  C     -0.815922      1.572735      0.673481
  C      0.596872      3.255024     -0.506567
  S     -0.525716      2.020660     -0.970245
  H      1.401171      4.031610      1.376489
  H     -0.115887      2.233926      2.636754
  H      1.083238      3.868159     -1.265628
  H     -1.495047      0.751217      0.899559
  C      2.247098     -2.141864      1.053435
  C      1.907269     -0.787136      1.346184
  C      2.088025      0.059733      0.256661
  C      2.678011     -2.309896     -0.261612
  S      2.673079     -0.805980     -1.122366
  H      2.179518     -2.968657      1.764280
  H      1.534763     -0.431364      2.309068
  H      2.999804     -3.224695     -0.759400
  H      1.905150      1.132150      0.189581
 ---------------------------------------------------

Cartesian coordinates of :   Thio3_ 8

 ---------------------------------------------------
 Atomic number (AN) and Cartesian coordinates
  AN         X             Y             Z
 ---------------------------------------------------
  C     -1.913022      1.500749      1.380140
  C     -3.167106      1.019835      0.897283
  C     -3.183709      0.878245     -0.488132
  C     -0.990548      1.716866      0.360572
  S     -1.666050      1.337753     -1.185806
  H     -1.681374      1.679558      2.432525
  H     -4.025159      0.778253      1.528431
  H      0.037322      2.071857      0.431717
  H     -3.998010      0.533191     -1.125496
  C      0.915382     -2.884347     -0.946543
  C      0.925085     -3.401025      0.383147
  C     -0.105802     -2.873044      1.161872
  C     -0.119508     -1.972273     -1.153239
  S     -1.073877     -1.751140      0.270359
  H      1.633930     -3.155674     -1.722964
  H      1.650547     -4.123019      0.764026
  H     -0.366339     -1.415923     -2.057214
  H     -0.341408     -3.087415      2.204655
  C      2.237843      1.032550      1.525808
  C      2.535937      2.282954      0.904216
  C      2.460282      2.214317     -0.486639
  C      1.933520      0.033489      0.604818
  S      2.027084      0.623795     -1.017855
  H      2.234022      0.863355      2.604757
  H      2.793227      3.198878      1.440730
  H      1.659101     -1.005674      0.786054
  H      2.639501      3.006187     -1.214209
 ---------------------------------------------------
```

Cartesian coordinates of : Thio3_ 9

Atomic number (AN) and Cartesian coordinates

AN	X	Y	Z
C	-3.122035	0.376884	0.254248
C	-4.189486	-0.535433	-0.002116
C	-3.737475	-1.829329	-0.246920
C	-1.870403	-0.229263	0.202749
S	-2.011280	-1.917851	-0.158778
H	-3.248117	1.439660	0.467882
H	-5.248230	-0.266688	-0.010239
H	-0.886632	0.212609	0.359524
H	-4.321235	-2.722407	-0.471127
C	1.414450	-1.783558	0.842755
C	2.637946	-1.449309	1.496732
C	3.602150	-0.968381	0.612511
C	1.465044	-1.553297	-0.530037
S	3.007806	-0.936208	-1.013894
H	0.528082	-2.173068	1.347584
H	2.815037	-1.550671	2.569658
H	0.686090	-1.711079	-1.275968
H	4.620976	-0.645629	0.827496
C	0.268666	2.266746	1.451305
C	-0.741880	3.012390	0.772697
C	-0.611225	2.944127	-0.612654
C	1.151542	1.640034	0.574731
S	0.745485	1.973399	-1.073996
H	0.349341	2.178047	2.536781
H	-1.535259	3.576143	1.268629
H	2.002331	0.999345	0.806831
H	-1.238312	3.404637	-1.376368

Cartesian coordinates of : Thio3_10

Atomic number (AN) and Cartesian coordinates

AN	X	Y	Z
C	0.766504	1.982685	-0.059090
C	0.521540	3.386578	0.014961
C	-0.837281	3.692279	0.042997
C	-0.409347	1.238365	-0.087194
S	-1.806708	2.258765	-0.024056
H	1.757226	1.524302	-0.089225
H	1.301692	4.150824	0.047535
H	-0.532978	0.159611	-0.153949
H	-1.311103	4.672810	0.099647
C	2.983071	-0.415015	1.401933
C	3.930390	-0.045685	0.400925
C	3.535809	-0.441444	-0.875613

```
  C      1.882295     -1.084080      0.873474
  S      2.012260     -1.261406     -0.843629
  H      3.085928     -0.191378      2.465608
  H      4.860621      0.492493      0.594880
  H      0.996102     -1.446927      1.392679
  H      4.056282     -0.296238     -1.822271
  C     -2.760859     -0.974813     -1.061773
  C     -1.682277     -1.851351     -1.383776
  C     -1.142761     -2.468793     -0.256782
  C     -3.024110     -0.932811      0.305634
  S     -1.959391     -1.974075      1.187957
  H     -3.317176     -0.382654     -1.791110
  H     -1.305003     -2.026359     -2.393359
  H     -3.775448     -0.347776      0.835978
  H     -0.332559     -3.196762     -0.204932
 ---------------------------------------------------



Cartesian coordinates of :   Thio4_ 1

 ---------------------------------------------------
 Atomic number (AN) and Cartesian coordinates
  AN          X             Y             Z
 ---------------------------------------------------
  C     -0.496274     -3.574136      0.679867
  C      0.648931     -3.395368     -0.152409
  C      1.466638     -2.351090      0.277313
  C     -0.532455     -2.662806      1.735184
  S      0.833204     -1.599991      1.700042
  H     -1.271880     -4.325401      0.516554
  H      0.867978     -3.991933     -1.040600
  H     -1.279500     -2.568442      2.523631
  H      2.386364     -1.971886     -0.169300
  C     -0.648884      3.395394     -0.152345
  C      0.496305      3.574077      0.679970
  C      0.532421      2.662697      1.735246
  C     -1.466646      2.351131      0.277308
  S     -0.833280      1.599941      1.700021
  H     -0.867890      3.992018     -1.040506
  H      1.271947      4.325316      0.516708
  H     -2.386386      1.972000     -0.169340
  H      1.279443      2.568268      2.523707
  C      3.196938     -0.454951     -2.125404
  C      1.841906     -0.006651     -2.135672
  C      1.515976      0.758235     -1.018112
  C      3.886282     -0.029455     -0.990679
  S      2.873653      0.926511      0.041386
  H      3.656290     -1.064279     -2.907019
  H      1.126646     -0.241256     -2.926222
  H      4.925976     -0.215681     -0.719846
  H      0.564772      1.224422     -0.760777
  C     -3.196923      0.454997     -2.125413
```

```
  C     -1.841884      0.006716     -2.135682
  C     -1.515948     -0.758182     -1.018131
  C     -3.886268      0.029470     -0.990698
  S     -2.873630     -0.926499      0.041354
  H     -3.656280      1.064332     -2.907018
  H     -1.126624      0.241348     -2.926223
  H     -4.925968      0.215670     -0.719872
  H     -0.564740     -1.224359     -0.760789
 ---------------------------------------------------

Cartesian coordinates of :   Thio4_ 2

 ---------------------------------------------------
 Atomic number (AN) and Cartesian coordinates
  AN         X             Y             Z
 ---------------------------------------------------
  C      3.774164      0.088667      1.158403
  C      3.328594     -0.490157     -0.067223
  C      2.110977     -1.156928      0.064222
  C      2.887851     -0.150204      2.208989
  S      1.523189     -1.081109      1.689023
  H      4.696345      0.661919      1.275771
  H      3.865907     -0.420903     -1.015592
  H      2.970324      0.168608      3.248343
  H      1.540767     -1.697192     -0.691337
  C     -3.328603      0.490135     -0.067251
  C     -3.774170     -0.088655      1.158392
  C     -2.887866      0.150265      2.208973
  C     -2.110994      1.156924      0.064174
  S     -1.523212      1.081170      1.688982
  H     -3.865908      0.420835     -1.015621
  H     -4.696350     -0.661907      1.275778
  H     -1.540801      1.697173     -0.691409
  H     -2.970341     -0.168504      3.248340
  C      0.088649     -3.774177     -1.158389
  C     -0.490118     -3.328586      0.067255
  C     -1.156887     -2.110967     -0.064178
  C     -0.150264     -2.887881     -2.208978
  S     -1.081141     -1.523205     -1.688992
  H      0.661889     -4.696366     -1.275770
  H     -0.420809     -3.865877      1.015632
  H      0.168492     -2.970374     -3.248348
  H     -1.697123     -1.540756      0.691401
  C     -0.088629      3.774150     -1.158447
  C      0.490105      3.328571      0.067217
  C      1.156874      2.110948     -0.064182
  C      0.150307      2.887840     -2.209018
  S      1.081168      1.523168     -1.688993
  H     -0.661847      4.696348     -1.275855
  H      0.420760      3.865867      1.015589
  H     -0.168394      2.970338     -3.248405
  H      1.697068      1.540733      0.691424
 ---------------------------------------------------
```

Cartesian coordinates of : Thio4_ 3

Atomic number (AN) and Cartesian coordinates

AN	X	Y	Z
C	-1.879213	1.444436	2.206194
C	-1.877952	0.104806	1.714522
C	-2.650743	-0.044722	0.565338
C	-2.652050	2.299451	1.421796
S	-3.379268	1.454486	0.097075
H	-1.344250	1.779877	3.098075
H	-1.331149	-0.726405	2.162549
H	-2.833004	3.366292	1.553889
H	-2.818392	-0.941675	-0.031596
C	1.612709	-0.190980	1.788088
C	1.527862	-1.583362	2.087226
C	2.419682	-2.346148	1.333514
C	2.569550	0.090742	0.815606
S	3.362080	-1.350781	0.275473
H	0.993282	0.584919	2.240486
H	0.847935	-2.023646	2.820326
H	2.834378	1.056851	0.384380
H	2.569601	-3.425854	1.346389
C	-2.006575	-2.186102	-2.188870
C	-0.884200	-1.313017	-2.064625
C	-0.109892	-1.586765	-0.939399
C	-2.071476	-3.116808	-1.152590
S	-0.754033	-2.919753	-0.043896
H	-2.740030	-2.143832	-2.997211
H	-0.648162	-0.511994	-2.768318
H	-2.807876	-3.905441	-0.995231
H	0.786638	-1.079057	-0.585089
C	1.973461	3.411780	-0.300868
C	0.767748	2.891361	0.257942
C	0.193470	1.896963	-0.530527
C	2.302113	2.806622	-1.513945
S	1.128910	1.614494	-1.958159
H	2.583146	4.195043	0.155121
H	0.327320	3.216459	1.202212
H	3.152211	3.009333	-2.165671
H	-0.701638	1.304458	-0.344591

Cartesian coordinates of : Thio4_ 4

Atomic number (AN) and Cartesian coordinates

AN	X	Y	Z
C	0.828182	-3.407092	0.077644
C	1.920062	-3.134410	-0.799560
C	1.561642	-2.279937	-1.841811
C	-0.343041	-2.754365	-0.302921

```
  S     -0.104242     -1.819300     -1.738157
  H      0.889666     -4.042624      0.963636
  H      2.929749     -3.532098     -0.677550
  H     -1.318716     -2.758280      0.184028
  H      2.183235     -1.911457     -2.658335
  C      0.532256      2.597057     -0.460651
  C      0.122281      1.769431     -1.546855
  C     -1.229738      1.915529     -1.852996
  C     -0.515553      3.364939      0.044051
  S     -1.990841      3.074828     -0.815481
  H      1.543363      2.622876     -0.049521
  H      0.785169      1.085363     -2.078823
  H     -0.499756      4.076506      0.869831
  H     -1.809555      1.414586     -2.628551
  C     -1.521658     -0.760634      2.173240
  C     -2.577311     -1.720121      2.220791
  C     -3.425301     -1.629027      1.117846
  C     -1.578506      0.052043      1.043316
  S     -2.929199     -0.362412      0.044140
  H     -0.741527     -0.673526      2.931706
  H     -2.717750     -2.450850      3.020433
  H     -0.904892      0.854517      0.740319
  H     -4.311233     -2.223445      0.892764
  C      1.827795      0.723006      2.022745
  C      2.916074      1.645042      1.983891
  C      3.715856      1.472617      0.855210
  C      1.811993     -0.142599      0.931306
  S      3.136687      0.174183     -0.135690
  H      1.075502      0.699209      2.813275
  H      3.112720      2.407061      2.741332
  H      1.105812     -0.940119      0.700317
  H      4.609542      2.026613      0.566656
 ---------------------------------------------------------

Cartesian coordinates of :   Thio4_ 5

 ---------------------------------------------------------
 Atomic number (AN) and Cartesian coordinates
  AN         X             Y             Z
 ---------------------------------------------------------
  C     -1.061860     -3.051461      2.083359
  C     -0.027123     -2.071489      2.177819
  C      0.646467     -1.882951      0.973199
  C     -1.157934     -3.598379      0.804609
  S      0.017482     -2.912916     -0.266703
  H     -1.715425     -3.346865      2.906937
  H      0.215434     -1.512590      3.084233
  H     -1.854421     -4.354584      0.442180
  H      1.467253     -1.204504      0.745156
  C      0.194608      3.598870     -0.128640
  C      1.353202      3.565818      0.704846
  C      1.271178      2.575738      1.683871
  C     -0.745448      2.630186      0.220276
  S     -0.212248      1.691623      1.571719
```

```
  H      0.048363      4.293723     -0.958531
  H      2.214021      4.229796      0.599781
  H     -1.711363      2.414886     -0.237037
  H      2.001783      2.325815      2.453543
  C     -3.898674      1.267033     -0.707295
  C     -3.435706      0.501192      0.405332
  C     -2.365662     -0.329850      0.078527
  C     -3.170932      1.006420     -1.868204
  S     -1.935608     -0.174389     -1.589386
  H     -4.724710      1.980624     -0.670412
  H     -3.859139      0.551094      1.410882
  H     -3.301785      1.439619     -2.860135
  H     -1.818917     -1.023328      0.716579
  C      2.793823     -1.440495     -1.738747
  C      1.718528     -0.520519     -1.930375
  C      1.816588      0.601984     -1.111702
  C      3.703642     -1.000787     -0.779458
  S      3.235869      0.533192     -0.120875
  H      2.903068     -2.387653     -2.271703
  H      0.893433     -0.669557     -2.629265
  H      4.609934     -1.494363     -0.428163
  H      1.136376      1.449859     -1.024611
 ---------------------------------------------------

Cartesian coordinates of :   Thio4_ 6

 ---------------------------------------------------
 Atomic number (AN) and Cartesian coordinates
  AN         X             Y             Z
 ---------------------------------------------------
  C     -0.619277     -3.554907     -0.138414
  C      0.519607     -3.667679     -0.990979
  C      0.627262     -2.593683     -1.874598
  C     -1.357749     -2.396841     -0.380297
  S     -0.661300     -1.458780     -1.655156
  H     -0.888905     -4.279281      0.633176
  H      1.239854     -4.488087     -0.960337
  H     -2.255475     -2.035344      0.122056
  H      1.387090     -2.419082     -2.636801
  C      3.085857      0.287571      2.280982
  C      1.853508     -0.433896      2.297117
  C      1.578831     -1.058898      1.082849
  C      3.734948      0.199948      1.050129
  S      2.836853     -0.770419     -0.069521
  H      3.486642      0.853031      3.124988
  H      1.179660     -0.489547      3.154644
  H      4.680617      0.651145      0.749413
  H      0.716199     -1.664499      0.805675
  C     -3.998778     -0.405032      0.910920
  C     -3.504509      0.303562     -0.225770
  C     -2.254573      0.877999      0.000686
  C     -3.112764     -0.359187      1.987459
  S     -1.691165      0.551162      1.602694
  H     -4.957209     -0.927527      0.949659
```

H	-4.034009	0.395506	-1.176638
H	-3.226795	-0.807587	2.974823
H	-1.649553	1.486267	-0.671071
C	-0.506875	3.827476	-0.794601
C	0.100788	3.275020	0.372003
C	0.918597	2.182599	0.087283
C	-0.138648	3.148284	-1.955802
S	0.949983	1.845862	-1.609556
H	-1.191904	4.678065	-0.794913
H	-0.054932	3.647618	1.386732
H	-0.444680	3.347828	-2.982942
H	1.506127	1.572273	0.772338

Cartesian coordinates of :   Thio4_ 7

Atomic number (AN) and Cartesian coordinates

AN	X	Y	Z
C	0.788521	3.362962	-0.353520
C	1.529238	2.252257	0.150599
C	1.040143	1.801362	1.374435
C	-0.252447	3.742091	0.492177
S	-0.314230	2.741022	1.903974
H	0.994004	3.864782	-1.301469
H	2.372713	1.782338	-0.358595
H	-0.980352	4.542437	0.359090
H	1.403707	0.972185	1.979986
C	1.741462	-0.306134	-2.149784
C	3.099281	0.125009	-2.240666
C	3.837607	-0.204337	-1.104987
C	1.461518	-0.958443	-0.951135
S	2.865968	-1.048822	0.055534
H	0.992282	-0.150152	-2.928731
H	3.527382	0.654391	-3.094847
H	0.522265	-1.395809	-0.613039
H	4.889788	-0.005894	-0.899241
C	-3.884170	0.077442	-0.613633
C	-2.831721	0.825864	-0.005717
C	-1.646767	0.782486	-0.738216
C	-3.487343	-0.526853	-1.806567
S	-1.834036	-0.167750	-2.171516
H	-4.892105	-0.022682	-0.204785
H	-2.919197	1.373799	0.934612
H	-4.076908	-1.145744	-2.483294
H	-0.683025	1.241121	-0.517116
C	-0.728475	-3.375252	0.378932
C	0.425399	-3.471477	1.212760
C	0.528245	-2.400538	2.100398
C	-1.483001	-2.231648	0.635455
S	-0.782483	-1.286670	1.903584
H	-0.996837	-4.099826	-0.392795
H	1.160065	-4.278130	1.166725

```
 H     -2.397184     -1.886836      0.150871
 H      1.294960     -2.219215      2.854227
 ---------------------------------------------------
```

Cartesian coordinates of :   Thio4_ 8

```
 ---------------------------------------------------
 Atomic number (AN) and Cartesian coordinates
  AN         X            Y            Z
 ---------------------------------------------------
 C     -3.561011     -1.452577      0.504710
 C     -2.848702     -0.372545     -0.097681
 C     -1.774321      0.063165      0.675427
 C     -3.019852     -1.826345      1.735037
 S     -1.650458     -0.848640      2.140185
 H     -4.433343     -1.943216      0.067149
 H     -3.098576      0.077804     -1.060284
 H     -3.358225     -2.606760      2.416937
 H     -1.053127      0.853947      0.468466
 C      0.725405     -3.439313     -0.222017
 C      1.842864     -3.110690     -1.046061
 C      1.528628     -2.148980     -2.006215
 C     -0.420391     -2.720464     -0.558954
 S     -0.126112     -1.654095     -1.888357
 H      0.751904     -4.158914      0.598983
 H      2.840070     -3.543591     -0.942602
 H     -1.409267     -2.745670     -0.099691
 H      2.177747     -1.718219     -2.769109
 C      0.526351      2.624669     -0.332822
 C      0.231699      1.931085     -1.543637
 C     -1.068371      2.160819     -1.990497
 C     -0.556155      3.374066      0.124196
 S     -1.922519      3.229473     -0.929666
 H      1.480001      2.569845      0.195750
 H      0.938080      1.282665     -2.064497
 H     -0.624306      3.993056      1.018978
 H     -1.559857      1.767427     -2.880743
 C      1.675792      0.496408      2.102460
 C      2.751963      1.430213      2.185266
 C      3.596107      1.366393      1.077511
 C      1.712259     -0.267043      0.937504
 S      3.071560      0.155507     -0.045921
 H      0.900702      0.380951      2.863073
 H      2.910595      2.123826      3.014058
 H      1.032437     -1.055442      0.615088
 H      4.492056      1.953208      0.873231
 ---------------------------------------------------
```

Cartesian coordinates of :   Thio4_ 9

```
 ---------------------------------------------------
 Atomic number (AN) and Cartesian coordinates
  AN         X            Y            Z
 ---------------------------------------------------
```

C	-1.720343	0.502173	-2.189979
C	-2.943513	1.233535	-2.260469
C	-3.810936	0.916755	-1.216503
C	-1.668484	-0.363572	-1.099650
S	-3.124236	-0.279095	-0.167634
H	-0.901939	0.616812	-2.902542
H	-3.186646	1.964132	-3.035136
H	-0.862775	-1.027986	-0.785709
H	-4.807608	1.312089	-1.018829
C	-0.940715	2.487276	0.401368
C	0.104969	3.375064	0.008363
C	1.238285	3.260427	0.812282
C	-0.589588	1.709007	1.501815
S	1.009689	2.074999	2.054272
H	-1.905560	2.398060	-0.102183
H	0.045221	4.067445	-0.834151
H	-1.184043	0.947130	2.005287
H	2.181150	3.803093	0.743368
C	1.720406	-0.502162	-2.189980
C	2.943574	-1.233532	-2.260433
C	3.810968	-0.916756	-1.216444
C	1.668520	0.363582	-1.099653
S	3.124247	0.279103	-0.167600
H	0.902011	-0.616811	-2.902553
H	3.186720	-1.964134	-3.035091
H	0.862814	1.028012	-0.785740
H	4.807629	-1.312100	-1.018735
C	0.940680	-2.487276	0.401354
C	-0.105004	-3.375063	0.008345
C	-1.238319	-3.260432	0.812264
C	0.589551	-1.709013	1.501806
S	-1.009723	-2.075012	2.054264
H	1.905529	-2.398062	-0.102188
H	-0.045250	-4.067448	-0.834166
H	1.184004	-0.947136	2.005280
H	-2.181186	-3.803094	0.743346

Cartesian coordinates of :   Thio4_10

Atomic number (AN) and Cartesian coordinates

AN	X	Y	Z
C	1.125672	-2.729410	0.246083
C	2.285801	-3.170383	-0.458553
C	2.372688	-2.617957	-1.736142
C	0.345717	-1.847366	-0.498237
S	1.030478	-1.574554	-2.063649
H	0.858608	-3.034178	1.259855
H	3.031133	-3.861577	-0.058855
H	-0.575032	-1.339307	-0.214649
H	3.137873	-2.781299	-2.495264
C	2.749933	0.285245	0.389512

```
  C      3.212982      1.557476     -0.061152
  C      2.752237      2.608525      0.730033
  C      1.941285      0.385525      1.519010
  S      1.765171      2.030036      2.030613
  H      2.975040     -0.669542     -0.089965
  H      3.848846      1.710484     -0.935712
  H      1.436933     -0.414089      2.059251
  H      2.939385      3.676691      0.620134
  C     -1.541657     -0.012273      1.871237
  C     -1.314386     -1.267522      2.510512
  C     -2.090468     -2.290930      1.967552
  C     -2.489181     -0.093902      0.853339
  S     -3.100905     -1.705362      0.689569
  H     -1.033792      0.920196      2.122680
  H     -0.619946     -1.427347      3.339215
  H     -2.844975      0.698595      0.194206
  H     -2.125113     -3.342644      2.252349
  C     -2.324744      2.962742     -0.893278
  C     -1.055148      2.771160     -0.270249
  C     -0.305391      1.762976     -0.872814
  C     -2.526538      2.094710     -1.966306
  S     -1.157129      1.063500     -2.207185
  H     -3.066885      3.699743     -0.578291
  H     -0.693354      3.337271      0.590412
  H     -3.390879      2.020791     -2.626640
  H      0.687512      1.396576     -0.611582
 ---------------------------------------------------

Cartesian coordinates of :   Thio4_11

 ---------------------------------------------------
 Atomic number (AN) and Cartesian coordinates
  AN         X             Y             Z
 ---------------------------------------------------
  C     -0.989325      0.687866      3.135806
  C     -1.371046     -0.035429      1.966868
  C     -0.388003     -0.929194      1.545839
  C      0.277006      0.329168      3.597677
  S      0.986844     -0.892704      2.597387
  H     -1.606928      1.444599      3.624784
  H     -2.319451      0.097419      1.444112
  H      0.818413      0.711488      4.463246
  H     -0.389662     -1.592126      0.679691
  C      0.270570     -1.779559     -2.100685
  C      0.271251     -3.065648     -1.482417
  C      1.337062     -3.226533     -0.597331
  C      1.320573     -0.972054     -1.664854
  S      2.319359     -1.803420     -0.525161
  H     -0.460754     -1.455437     -2.844681
  H     -0.469442     -3.845516     -1.671589
  H      1.572215      0.050683     -1.949992
  H      1.590425     -4.097660      0.007201
  C     -4.010658      1.255142     -0.247874
  C     -2.736096      1.656294     -0.748841
```

```
  C     -1.990848      0.583838     -1.231148
  C     -4.216461     -0.119402     -0.359253
  S     -2.852466     -0.908459     -1.078006
  H     -4.751580      1.932623      0.182057
  H     -2.363617      2.682997     -0.749684
  H     -5.090481     -0.700175     -0.064327
  H     -0.978578      0.587703     -1.627681
  C      3.251212      1.845433     -0.849745
  C      2.613178      1.436938      0.359620
  C      1.271206      1.807417      0.414678
  C      2.386157      2.527613     -1.704664
  S      0.805103      2.670966     -1.011679
  H      4.297173      1.650274     -1.096907
  H      3.100581      0.882582      1.163605
  H      2.596498      2.959683     -2.683114
  H      0.539067      1.617393      1.200558
 ---------------------------------------------------

Cartesian coordinates of :   Thio4_12

 ---------------------------------------------------
 Atomic number (AN) and Cartesian coordinates
  AN         X            Y             Z
 ---------------------------------------------------
  C      1.950404     -3.398264      0.910950
  C      2.616575     -2.586180     -0.056009
  C      2.445125     -1.222426      0.178379
  C      1.287754     -2.636267      1.872757
  S      1.478984     -0.940905      1.584270
  H      1.949848     -4.490470      0.910321
  H      3.195978     -2.974248     -0.896684
  H      0.707489     -2.984964      2.727612
  H      2.830736     -0.374634     -0.388140
  C     -1.393764      1.273774      2.277858
  C     -1.495469      2.697951      2.258348
  C     -1.944044      3.180268      1.029496
  C     -1.761268      0.690654      1.067157
  S     -2.239886      1.889073     -0.085875
  H     -1.052886      0.689599      3.135284
  H     -1.249200      3.350425      3.098797
  H     -1.771413     -0.363393      0.790534
  H     -2.107635      4.214494      0.726389
  C     -3.086948     -2.375729     -0.264811
  C     -1.739812     -2.742856      0.030766
  C     -0.815513     -2.119124     -0.806079
  C     -3.171088     -1.477683     -1.327902
  S     -1.603944     -1.096629     -1.957665
  H     -3.963290     -2.738895      0.276122
  H     -1.439194     -3.427350      0.826137
  H     -4.061411     -1.027050     -1.766841
  H      0.271940     -2.194600     -0.798087
  C      2.647415      2.208469      0.236882
  C      3.439195      2.003429     -0.933284
  C      2.670675      1.612994     -2.029821
```

```
  C      1.291399      1.972909      0.016494
  S      0.990692      1.507213     -1.622530
  H      3.044459      2.512019      1.208051
  H      4.523032      2.129935     -0.980581
  H      0.460818      2.056508      0.716941
  H      3.003378      1.391068     -3.044121
 ---------------------------------------------------
```

Cartesian coordinates of :   Thio4_13

```
 ---------------------------------------------------
 Atomic number (AN) and Cartesian coordinates
  AN          X             Y             Z
 ---------------------------------------------------
  C      2.932473     -1.097548     -0.229220
  C      3.802807     -0.041844     -0.635398
  C      3.259741      0.725855     -1.665268
  C      1.738284     -1.118555     -0.947757
  S      1.697385      0.145049     -2.128286
  H      3.152538     -1.817424      0.561246
  H      4.784500      0.157163     -0.200084
  H      0.884312     -1.786524     -0.833313
  H      3.701912      1.579781     -2.179186
  C     -0.041791     -3.802773      0.635445
  C     -1.097535     -2.932498      0.229248
  C     -1.118602     -1.738299      0.947766
  C      0.725878     -3.259655      1.665310
  S      0.144993     -1.697321      2.128302
  H      0.157265     -4.784467      0.200152
  H     -1.817407     -3.152621     -0.561206
  H      1.579826     -3.701774      2.179236
  H     -1.786622     -0.884367      0.833314
  C      1.097571      2.932476      0.229289
  C      0.041824      3.802783      0.635410
  C     -0.725921      3.259697      1.665236
  C      1.118568      1.738288      0.947827
  S     -0.145101      1.697356      2.128287
  H      1.817496      3.152569     -0.561126
  H     -0.157184      4.784473      0.200088
  H      1.786575      0.884340      0.833433
  H     -1.579892      3.701843      2.179101
  C     -3.802807      0.041753     -0.635538
  C     -2.932557      1.097498     -0.229288
  C     -1.738319      1.118576     -0.947739
  C     -3.259627     -0.725908     -1.665376
  S     -1.697271     -0.145013     -2.128281
  H     -4.784519     -0.157312     -0.200292
  H     -3.152722      1.817359      0.561165
  H     -3.701713     -1.579854     -2.179335
  H     -0.884395      1.786596     -0.833231
 ---------------------------------------------------
```

Cartesian coordinates of :   Thio4_14

```
 ---------------------------------------------------
 Atomic number (AN) and Cartesian coordinates
  AN          X             Y             Z
 ---------------------------------------------------
  C      0.649616     -3.223152     -1.899551
  C     -0.203311     -2.088191     -2.053986
  C     -0.933834     -1.804234     -0.902356
  C      0.552666     -3.789545     -0.629207
  S     -0.582239     -2.933468      0.359577
  H      1.311933     -3.613443     -2.675206
  H     -0.277980     -1.488936     -2.963960
  H      1.086892     -4.651552     -0.229561
  H     -1.648335     -1.002133     -0.724358
  C      0.234917      3.585702      0.183731
  C     -0.856850      3.719386     -0.726717
  C     -0.872263      2.705955     -1.684814
  C      1.027179      2.471227     -0.088542
  S      0.439811      1.599739     -1.461341
  H      0.436970      4.267376      1.012841
  H     -1.603255      4.515849     -0.690035
  H      1.908947      2.113359      0.443047
  H     -1.579657      2.559832     -2.501445
  C      1.866590     -0.428397      1.592883
  C      2.842140      0.421973      2.192903
  C      3.872715      0.754068      1.313418
  C      2.162704     -0.734924      0.267122
  S      3.640742      0.013960     -0.235557
  H      0.973323     -0.808181      2.090820
  H      2.801833      0.786136      3.221939
  H      1.604684     -1.358011     -0.430603
  H      4.747221      1.378828      1.495868
  C     -1.983103     -0.199570      1.934106
  C     -3.135781     -1.003287      1.679213
  C     -3.914036     -0.497725      0.640029
  C     -1.891006      0.898959      1.083287
  S     -3.228185      0.952848     -0.016183
  H     -1.245522     -0.409164      2.711360
  H     -3.392010     -1.913643      2.225586
  H     -1.117086      1.665359      1.031284
  H     -4.840066     -0.900424      0.229378
 ---------------------------------------------------

Cartesian coordinates of :   Thio4_15

 ---------------------------------------------------
 Atomic number (AN) and Cartesian coordinates
  AN          X             Y             Z
 ---------------------------------------------------
  C     -0.796176      2.452792     -0.369392
  C     -1.587902      3.284244      0.477602
  C     -2.854092      2.749025      0.710463
  C     -1.462140      1.295190     -0.765456
  S     -3.059283      1.235973     -0.105722
  H      0.234352      2.666403     -0.655978
```

```
  H     -1.253822      4.230839      0.907989
  H     -1.100830      0.471623     -1.382160
  H     -3.665509      3.164063      1.308799
  C     -0.758973     -0.759011      3.131043
  C     -0.473256      0.295216      2.212819
  C      0.716593      0.090845      1.519481
  C      0.215988     -1.756631      3.122551
  S      1.483096     -1.387326      1.998182
  H     -1.642293     -0.799614      3.772788
  H     -1.109501      1.167144      2.046451
  H      0.259242     -2.669978      3.716496
  H      1.181932      0.720100      0.762643
  C      3.631841      1.696616      0.002823
  C      3.806076      0.284889      0.114865
  C      3.087249     -0.419145     -0.848909
  C      2.791705      2.045804     -1.052971
  S      2.210654      0.647296     -1.892831
  H      4.096577      2.432983      0.661409
  H      4.419419     -0.205567      0.873387
  H      2.501204      3.042274     -1.386264
  H      3.021827     -1.497054     -0.995095
  C     -2.564498     -1.958029     -0.691416
  C     -2.494996     -1.826148     -2.111046
  C     -1.185577     -1.891488     -2.586455
  C     -1.311816     -2.112908     -0.102488
  S     -0.057162     -2.115003     -1.291676
  H     -3.489399     -1.930714     -0.111893
  H     -3.358442     -1.684350     -2.764736
  H     -1.057681     -2.200423      0.954365
  H     -0.834112     -1.830341     -3.616551
 ---------------------------------------------------

Cartesian coordinates of :   Thio4_16

 ---------------------------------------------------
 Atomic number (AN) and Cartesian coordinates
  AN          X             Y             Z
 ---------------------------------------------------
  C     -3.831137     -1.428985      0.549981
  C     -2.511865     -1.574140      1.074928
  C     -1.929867     -0.350958      1.397172
  C     -4.233465     -0.095764      0.480615
  S     -2.997134      0.968922      1.063301
  H     -4.467096     -2.257392      0.230326
  H     -1.999786     -2.529563      1.209888
  H     -5.183114      0.309306      0.130723
  H     -0.937406     -0.144950      1.793319
  C     -1.087922     -0.991556     -2.973457
  C      0.210603     -0.580925     -3.401014
  C      0.673658      0.528208     -2.694496
  C     -1.590125     -0.187335     -1.950988
  S     -0.474692      1.062705     -1.517160
  H     -1.637542     -1.840417     -3.386304
  H      0.789125     -1.072072     -4.186997
```

```
  H     -2.544642     -0.269598     -1.430708
  H      1.625278      1.048422     -2.795234
  C      2.604434      2.070019     -0.320893
  C      1.802591      3.245307     -0.202310
  C      0.934614      3.187907      0.886586
  C      2.332461      1.129336      0.669446
  S      1.112131      1.700606      1.752318
  H      3.358476      1.909336     -1.094918
  H      1.850237      4.102207     -0.877978
  H      2.778992      0.146560      0.821288
  H      0.212732      3.933898      1.219160
  C      3.293063     -2.291687      0.159319
  C      2.289368     -1.989143     -0.809396
  C      1.024151     -1.839126     -0.247266
  C      2.779388     -2.368860      1.453515
  S      1.071416     -2.083036      1.466369
  H      4.350505     -2.445246     -0.067462
  H      2.470341     -1.870695     -1.878842
  H      3.305480     -2.588778      2.382490
  H      0.081199     -1.591958     -0.736435
 ---------------------------------------------------

Cartesian coordinates of :   Thio4_17

 ---------------------------------------------------
 Atomic number (AN) and Cartesian coordinates
  AN          X             Y             Z
 ---------------------------------------------------
  C      1.081476      1.783010     -1.931503
  C      2.413669      1.762323     -2.443048
  C      3.369261      1.754943     -1.427153
  C      1.036476      1.780337     -0.537920
  S      2.627964      1.773117      0.137311
  H      0.187402      1.797764     -2.557826
  H      2.671954      1.754524     -3.504182
  H      0.169509      1.777470      0.124510
  H      4.455490      1.743303     -1.520579
  C      0.475059      1.524785      2.927890
  C     -0.847172      1.853203      2.501590
  C     -1.454902      0.810574      1.802921
  C      0.847050      0.234404      2.550121
  S     -0.411171     -0.562985      1.671924
  H      1.134754      2.197168      3.481016
  H     -1.338121      2.811399      2.685493
  H      1.789519     -0.280590      2.732737
  H     -2.447486      0.781947      1.353822
  C     -1.900329      0.066194     -1.366845
  C     -2.526615      1.346909     -1.376606
  C     -3.826550      1.309747     -0.875436
  C     -2.730765     -0.929494     -0.859570
  S     -4.274824     -0.294597     -0.399187
  H     -0.875524     -0.130953     -1.681134
  H     -2.053658      2.270046     -1.718830
  H     -2.509670     -1.988456     -0.725444
```

```
  H     -4.536825      2.129178     -0.764112
  C      2.489934     -1.852114      0.398645
  C      1.856529     -2.995504      0.970573
  C      0.821363     -3.483604      0.174662
  C      1.929598     -1.478705     -0.820931
  S      0.640162     -2.542840     -1.265556
  H      3.323414     -1.310062      0.850191
  H      2.137639     -3.452740      1.922027
  H      2.202538     -0.646265     -1.470540
  H      0.170461     -4.338885      0.356643
 ---------------------------------------------------

Cartesian coordinates of :   Thio4_18

 ---------------------------------------------------
 Atomic number (AN) and Cartesian coordinates
  AN         X             Y             Z
 ---------------------------------------------------
  C      0.931479      1.386896     -1.588405
  C      0.131338      2.235220     -2.410518
  C     -0.119374      3.472619     -1.816621
  C      1.279452      1.985525     -0.380182
  S      0.636133      3.588634     -0.262678
  H      1.236646      0.372575     -1.847424
  H     -0.250697      1.965029     -3.397943
  H      1.873156      1.573102      0.435931
  H     -0.689731      4.313559     -2.211445
  C     -2.235233      0.131330      2.410505
  C     -1.386900      0.931452      1.588383
  C     -1.985529      1.279426      0.380161
  C     -3.472638     -0.119367      1.816615
  S     -3.588648      0.636130      0.262668
  H     -1.965046     -0.250690      3.397937
  H     -0.372568      1.236591      1.847390
  H     -4.313585     -0.689708      2.211448
  H     -1.573098      1.873114     -0.435959
  C     -0.931399     -1.386877     -1.588404
  C     -0.131285     -2.235237     -2.410507
  C      0.119364     -3.472647     -1.816607
  C     -1.279415     -1.985491     -0.380187
  S     -0.636170     -3.588629     -0.262674
  H     -1.236494     -0.372533     -1.847417
  H      0.250776     -1.965061     -3.397926
  H     -1.873102     -1.573033      0.435919
  H      0.689686     -4.313614     -2.211426
  C      2.235195     -0.131299      2.410506
  C      1.386875     -0.931439      1.588387
  C      1.985527     -1.279445      0.380186
  C      3.472613      0.119379      1.816635
  S      3.588650     -0.636157      0.262708
  H      1.964986      0.250758      3.397917
  H      0.372540     -1.236575      1.847386
  H      4.313554      0.689729      2.211469
  H      1.573113     -1.873157     -0.435927
```

Cartesian coordinates of :   Thio4_19

Atomic number (AN) and Cartesian coordinates

AN	X	Y	Z
C	-4.101536	-1.095853	-0.625374
C	-2.923073	-1.214370	-1.421857
C	-2.188818	-0.031784	-1.467617
C	-4.243559	0.177170	-0.074545
S	-2.943971	1.219570	-0.542984
H	-4.817815	-1.901459	-0.450996
H	-2.610929	-2.125900	-1.936170
H	-5.041039	0.552818	0.566626
H	-1.246302	0.160890	-1.976113
C	1.882699	2.198504	1.208289
C	2.633872	2.889531	0.210393
C	1.921838	3.046914	-0.978464
C	0.609455	1.838126	0.773412
S	0.337163	2.360133	-0.853841
H	2.250900	1.958365	2.207516
H	3.653564	3.257744	0.343204
H	-0.169173	1.291110	1.306427
H	2.236517	3.535826	-1.900428
C	2.947574	-1.657821	-0.380401
C	2.407038	-2.864792	-0.917147
C	1.483461	-2.627128	-1.934637
C	2.425822	-0.517217	-0.986713
S	1.294293	-0.930705	-2.226561
H	3.682947	-1.613884	0.425524
H	2.672321	-3.868579	-0.578401
H	2.633263	0.531357	-0.771134
H	0.926999	-3.352273	-2.528628
C	-1.229407	-0.414845	2.960573
C	-1.311358	-0.993045	1.659005
C	-0.077093	-1.444754	1.195789
C	0.066458	-0.443444	3.476733
S	1.170348	-1.176972	2.363839
H	-2.076720	0.010276	3.503431
H	-2.228986	-1.069596	1.074504
H	0.420119	-0.081835	4.442758
H	0.168786	-1.908446	0.240006

Cartesian coordinates of :   Thio4_20

Atomic number (AN) and Cartesian coordinates

AN	X	Y	Z
C	-0.789899	-0.924496	3.091529
C	-0.417787	0.149007	2.227890

C	0.725124	-0.135739	1.486086
C	0.073253	-2.016038	2.992695
S	1.341438	-1.713305	1.849316
H	-1.654982	-0.912313	3.758808
H	-0.966629	1.088233	2.130130
H	0.034876	-2.963575	3.530778
H	1.237297	0.492644	0.759630
C	-2.871888	-1.845844	-0.689916
C	-2.871855	-1.650457	-2.103666
C	-1.587777	-1.701280	-2.645197
C	-1.593476	-2.033314	-0.171016
S	-0.398419	-1.987492	-1.418905
H	-3.767419	-1.843920	-0.064577
H	-3.764780	-1.476176	-2.708028
H	-1.288420	-2.172136	0.866436
H	-1.287672	-1.596439	-3.687982
C	3.753319	-0.225004	-0.084144
C	3.722959	1.190631	0.096630
C	2.852600	1.821493	-0.789089
C	2.900989	-0.650423	-1.100529
S	2.075851	0.682005	-1.836476
H	4.361510	-0.912810	0.506840
H	4.301218	1.732084	0.847931
H	2.715589	-1.665798	-1.449914
H	2.624662	2.883290	-0.878136
C	-2.868767	1.487485	0.117624
C	-2.846771	2.622864	0.981253
C	-1.663790	3.352255	0.870272
C	-1.707029	1.362590	-0.639911
S	-0.596442	2.644049	-0.294532
H	-3.690109	0.772633	0.051164
H	-3.652704	2.900470	1.664274
H	-1.445245	0.588938	-1.361812
H	-1.371295	4.258364	1.400946

Cartesian coordinates of :   Thio4_21

Atomic number (AN) and Cartesian coordinates

AN	X	Y	Z
C	4.806589	-0.829150	-0.061701
C	4.294640	0.239151	0.734946
C	3.286157	0.950837	0.089910
C	4.178922	-0.912013	-1.303998
S	2.973526	0.315783	-1.487907
H	5.598132	-1.513627	0.251147
H	4.641902	0.481936	1.741484
H	4.369216	-1.616873	-2.113623
H	2.704407	1.797528	0.453555
C	-0.403422	-1.280597	2.163119
C	1.021287	-1.256835	2.069704
C	1.488863	-1.802286	0.874655

```
  C     -1.006102     -1.838346      1.037544
  S      0.177734     -2.343616     -0.120442
  H     -0.977804     -0.893498      3.007638
  H      1.689047     -0.847665      2.831345
  H     -2.067398     -1.960074      0.818716
  H      2.517173     -1.894867      0.523719
  C     -4.443084     -1.418885      0.042599
  C     -4.101232     -0.156922      0.616384
  C     -3.185714      0.553221     -0.156826
  C     -3.783438     -1.647795     -1.165065
  S     -2.751729     -0.322438     -1.582154
  H     -5.137817     -2.135886      0.485629
  H     -4.502189      0.223603      1.558329
  H     -3.853173     -2.516354     -1.820411
  H     -2.749320      1.535359      0.025891
  C     -0.866300      3.296461     -0.754828
  C     -0.281114      2.044145     -1.113419
  C      0.013375      1.253424     -0.005019
  C     -0.997591      3.444223      0.625933
  S     -0.408040      2.049086      1.469702
  H     -1.182367      4.062058     -1.466768
  H     -0.089824      1.717437     -2.138268
  H     -1.399450      4.292869      1.179918
  H      0.444610      0.255225      0.030878
 ---------------------------------------------------

Cartesian coordinates of :   Thio4_22

 ---------------------------------------------------
 Atomic number (AN) and Cartesian coordinates
  AN          X             Y             Z
 ---------------------------------------------------
  C     -0.665173      1.424077      1.971055
  C     -1.892236      1.920671      2.503415
  C     -2.682219      2.530865      1.529492
  C     -0.537175      1.650921      0.601105
  S     -1.914795      2.489445     -0.022512
  H      0.093126      0.909879      2.563958
  H     -2.194606      1.837808      3.549607
  H      0.263404      1.347957     -0.074885
  H     -3.658293      3.002015      1.646584
  C     -2.408795     -3.130075     -0.540757
  C     -3.013534     -1.838163     -0.483273
  C     -2.489959     -1.044419      0.534782
  C     -1.433729     -3.303524      0.441547
  S     -1.267073     -1.885423      1.420241
  H     -2.666347     -3.905256     -1.265764
  H     -3.796855     -1.490094     -1.160082
  H     -0.820221     -4.182892      0.638883
  H     -2.750146     -0.022974      0.812952
  C      1.381590      0.977990     -2.349886
  C      1.323744     -0.293687     -1.705241
  C      0.047645     -0.852276     -1.740891
  C      0.148201      1.366499     -2.875020
```

```
  S     -1.068194      0.171253     -2.575250
  H      2.282896      1.590266     -2.427134
  H      2.169516     -0.781201     -1.217576
  H     -0.101091      2.277054     -3.420637
  H     -0.301977     -1.794318     -1.317638
  C      3.214068     -1.599590      1.009019
  C      2.324697     -0.627414      1.554567
  C      2.695986      0.675829      1.231491
  C      4.249276     -1.023439      0.274492
  S      4.130374      0.703551      0.265273
  H      3.106486     -2.678901      1.136528
  H      1.439158     -0.871190      2.142795
  H      5.066915     -1.516662     -0.251223
  H      2.208737      1.611699      1.504213
 ---------------------------------------------------
```

```
Cartesian coordinates of :   Thio4_23

 ---------------------------------------------------
 Atomic number (AN) and Cartesian coordinates
  AN         X             Y             Z
 ---------------------------------------------------
  C      4.413870      0.538284      0.375751
  C      4.783363     -0.715177     -0.198076
  C      3.967862     -1.069652     -1.272148
  C      3.324626      1.120363     -0.268755
  S      2.764173      0.134567     -1.574696
  H      4.916176      1.001792      1.227335
  H      5.607615     -1.340005      0.152606
  H      2.818402      2.059151     -0.044625
  H      4.020068     -1.959737     -1.899316
  C     -1.975377     -0.792742     -1.697091
  C     -2.460852     -2.126830     -1.549599
  C     -3.598690     -2.193594     -0.746508
  C     -2.748598      0.138890     -1.010759
  S     -4.067742     -0.621401     -0.188054
  H     -1.085650     -0.512844     -2.264926
  H     -2.001794     -3.008972     -2.001835
  H     -2.608460      1.217560     -0.937696
  H     -4.181761     -3.070763     -0.465434
  C     -1.057300      3.284564     -0.972102
  C     -0.082689      2.241949     -0.991267
  C     -0.027444      1.533371      0.207922
  C     -1.726715      3.359489      0.249726
  S     -1.159306      2.154457      1.356138
  H     -1.269941      3.952704     -1.809549
  H      0.545332      2.004413     -1.853202
  H     -2.509423      4.056617      0.550115
  H      0.607766      0.691568      0.481429
  C     -0.335259     -0.886011      2.565400
  C      1.090162     -0.800351      2.551897
  C      1.648559     -1.374430      1.409945
  C     -0.844601     -1.517654      1.432068
  S      0.424309     -2.015659      0.367782
```

```
  H     -0.972940    -0.493542     3.360636
  H      1.691367    -0.330888     3.333815
  H     -1.882530    -1.700398     1.153776
  H      2.697321    -1.440885     1.119729
 ---------------------------------------------------

Cartesian coordinates of :   Thio4_24

 ---------------------------------------------------
 Atomic number (AN) and Cartesian coordinates
  AN          X            Y            Z
 ---------------------------------------------------
  C      2.120365     0.442400    -1.972194
  C      2.645923    -0.836104    -1.620942
  C      3.646031    -0.752690    -0.653379
  C      2.726857     1.479849    -1.266105
  S      3.939171     0.886327    -0.182592
  H      1.323357     0.608327    -2.699286
  H      2.305648    -1.783581    -2.043926
  H      2.528823     2.549907    -1.330132
  H      4.217711    -1.560263    -0.196125
  C     -3.262199    -1.851567    -0.454679
  C     -3.534500    -1.460310    -1.800900
  C     -2.372049    -1.152973    -2.507297
  C     -1.902810    -1.831710    -0.153027
  S     -0.961858    -1.345265    -1.519229
  H     -4.025693    -2.128127     0.275675
  H     -4.531796    -1.400779    -2.242085
  H     -1.409076    -2.055090     0.792743
  H     -2.276396    -0.834815    -3.545727
  C     -1.904183     3.184122     1.504233
  C     -2.810519     2.188142     1.030362
  C     -2.349095     1.543132    -0.114617
  C     -0.762641     3.283979     0.709047
  S     -0.811777     2.160547    -0.606893
  H     -2.066334     3.801754     2.390248
  H     -3.760689     1.938556     1.507387
  H      0.091202     3.950726     0.831321
  H     -2.815868     0.738888    -0.683455
  C     -0.523652    -1.032508     3.012863
  C     -0.163728     0.020936     2.120514
  C      0.821046    -0.361830     1.213897
  C      0.188925    -2.207257     2.770894
  S      1.305017    -2.007247     1.458985
  H     -1.277744    -0.948548     3.798916
  H     -0.613497     1.015807     2.116406
  H      0.123367    -3.161609     3.293970
  H      1.275493     0.225399     0.417713
 ---------------------------------------------------

Cartesian coordinates of :   Thio4_25

 ---------------------------------------------------
 Atomic number (AN) and Cartesian coordinates
```

AN	X	Y	Z
C	2.289128	-1.909516	0.071447
C	2.780529	-2.215052	-1.232954
C	1.763135	-2.565512	-2.118585
C	0.905390	-2.026509	0.168520
S	0.216767	-2.529297	-1.339130
H	2.907830	-1.597156	0.914744
H	3.832432	-2.180041	-1.525850
H	0.260677	-1.826084	1.024951
H	1.840912	-2.851032	-3.167565
C	-4.008861	0.864254	0.075551
C	-3.846180	-0.520992	0.375339
C	-2.885047	-1.131286	-0.427993
C	-3.173693	1.284291	-0.958556
S	-2.197659	-0.012920	-1.554595
H	-4.700152	1.534009	0.591212
H	-4.402161	-1.056795	1.147757
H	-3.079699	2.279137	-1.393806
H	-2.562854	-2.172424	-0.426895
C	-0.831805	0.551058	1.753786
C	-1.159523	-0.511627	2.648893
C	-0.073319	-0.915258	3.424738
C	0.498483	0.947217	1.863217
S	1.339509	0.018270	3.057872
H	-1.530742	1.003812	1.047961
H	-2.147262	-0.970034	2.724364
H	1.032982	1.718128	1.308400
H	-0.031452	-1.695085	4.185370
C	2.741928	2.716649	-0.404643
C	2.672118	1.356000	-0.828214
C	1.423367	1.014200	-1.341644
C	1.541942	3.398130	-0.606687
S	0.344595	2.366768	-1.313517
H	3.624446	3.187029	0.034530
H	3.491270	0.639037	-0.754104
H	1.304807	4.438030	-0.381776
H	1.082024	0.048056	-1.712656

Cartesian coordinates of :   Thio4_26

Atomic number (AN) and Cartesian coordinates

AN	X	Y	Z
C	-0.555539	-1.179852	2.955326
C	-0.206660	-0.061226	2.141553
C	0.836710	-0.345098	1.264246
C	0.223200	-2.304996	2.683133
S	1.388771	-1.977724	1.442033
H	-1.347885	-1.176015	3.707465
H	-0.703092	0.910712	2.171838
H	0.180597	-3.290353	3.147759

```
  H      1.305708      0.310915      0.532169
  C      2.570034      1.854765     -1.090991
  C      2.083178      0.778883     -1.891508
  C      2.738625     -0.421380     -1.625676
  C      3.589222      1.457547     -0.227118
  S      3.950763     -0.224984     -0.405612
  H      2.195069      2.879541     -1.137453
  H      1.278486      0.867897     -2.623323
  H      4.138761      2.058013      0.497746
  H      2.570212     -1.400469     -2.074446
  C     -2.057736      3.049525      1.603704
  C     -2.924256      2.037922      1.089931
  C     -2.426863      1.441528     -0.066481
  C     -0.910068      3.209449      0.827114
  S     -0.905347      2.122938     -0.519920
  H     -2.251685      3.635760      2.504548
  H     -3.871414      1.743602      1.547063
  H     -0.079968      3.899503      0.979851
  H     -2.857686      0.637680     -0.663674
  C     -3.420129     -1.536829     -1.915274
  C     -3.142138     -1.979074     -0.586150
  C     -1.788890     -1.903447     -0.266016
  C     -2.267919     -1.134960     -2.590359
  S     -0.860261     -1.301971     -1.593962
  H     -4.414339     -1.507699     -2.366373
  H     -3.897811     -2.330217      0.119883
  H     -2.178055     -0.761416     -3.610712
  H     -1.294343     -2.146134      0.674427
 ---------------------------------------------------

Cartesian coordinates of :   Thio4_27

 ---------------------------------------------------
 Atomic number (AN) and Cartesian coordinates
  AN          X             Y             Z
 ---------------------------------------------------
  C     -4.053988     -0.224488      0.892737
  C     -4.553745     -1.345101      0.162752
  C     -4.089771     -1.370726     -1.152151
  C     -3.214893      0.582950      0.129198
  S     -3.048358     -0.026677     -1.480758
  H     -4.286208     -0.013443      1.938768
  H     -5.220520     -2.107108      0.572117
  H     -2.687451      1.490322      0.423139
  H     -4.305399     -2.100497     -1.932722
  C     -0.424981     -2.124814      1.850490
  C      1.001060     -2.090538      1.779222
  C      1.467711     -1.936195      0.474466
  C     -1.027340     -1.994734      0.601183
  S      0.156170     -1.841596     -0.652810
  H     -0.998603     -2.227662      2.774257
  H      1.669376     -2.166120      2.639939
  H     -2.088704     -1.974141      0.354887
  H      2.497249     -1.869827      0.122399
```

```
  C     -0.535033      2.137190      1.848686
  C     -1.044576      3.312313      1.216267
  C     -0.862352      3.290694     -0.166025
  C      0.022314      1.237103      0.944005
  S     -0.072125      1.835357     -0.677131
  H     -0.580428      1.942690      2.922462
  H     -1.526085      4.141436      1.739298
  H      0.470968      0.262881      1.137769
  H     -1.147621      4.045943     -0.898567
  C      4.263087      0.079966      0.996649
  C      4.855165     -0.854495      0.094919
  C      4.368240     -0.718817     -1.204617
  C      3.335073      0.910302      0.372780
  S      3.195616      0.549847     -1.313465
  H      4.497110      0.146411      2.061241
  H      5.603359     -1.599505      0.374273
  H      2.724442      1.700860      0.807972
  H      4.641833     -1.286808     -2.093982
 ---------------------------------------------------

Cartesian coordinates of :   Thio4_28

 ---------------------------------------------------
 Atomic number (AN) and Cartesian coordinates
  AN         X             Y             Z
 ---------------------------------------------------
  C     -4.068230      0.146909      0.394319
  C     -4.290338     -0.998985     -0.425268
  C     -3.461449     -1.018147     -1.546501
  C     -3.073372      0.980990     -0.111923
  S     -2.421788      0.363250     -1.590767
  H     -4.607954      0.361002      1.319140
  H     -5.019680     -1.783304     -0.212316
  H     -2.707372      1.923597      0.295044
  H     -3.411390     -1.763697     -2.340155
  C     -1.366726     -1.042559      2.503461
  C     -1.154271     -1.499930      1.168479
  C      0.189463     -1.740582      0.893954
  C     -0.179605     -0.948328      3.230274
  S      1.184143     -1.420688      2.272360
  H     -2.343473     -0.788322      2.920784
  H     -1.945338     -1.636840      0.429615
  H     -0.040551     -0.635419      4.265426
  H      0.645313     -2.072912     -0.038528
  C      2.228007      3.075193      0.734103
  C      1.553497      2.080361      1.503710
  C      0.332654      1.703483      0.948816
  C      1.510264      3.448828     -0.401543
  S      0.015512      2.582227     -0.508916
  H      3.199734      3.504517      0.987772
  H      1.942847      1.635380      2.420970
  H      1.774537      4.182032     -1.163482
  H     -0.374352      0.948623      1.295238
  C      1.406630     -1.831053     -2.459750
```

C	1.268525	-0.444616	-2.150690
C	2.221408	-0.001501	-1.237211
C	2.464350	-2.427630	-1.773795
S	3.283848	-1.282891	-0.764293
H	0.763172	-2.381557	-3.149872
H	0.498264	0.210985	-2.560115
H	2.803083	-3.462981	-1.813567
H	2.350855	0.993945	-0.812731

Cartesian coordinates of : Thio4_29

Atomic number (AN) and Cartesian coordinates

AN	X	Y	Z
C	-0.753713	2.003415	1.529949
C	-1.121710	3.237231	0.911819
C	-0.703963	3.308595	-0.416895
C	-0.066089	1.153122	0.667106
S	0.131360	1.867392	-0.896140
H	-0.986105	1.733675	2.562660
H	-1.669088	4.042292	1.406977
H	0.321125	0.152641	0.859052
H	-0.840440	4.125236	-1.126146
C	4.848687	-0.906304	0.574552
C	4.070480	-0.068989	1.429044
C	3.285201	0.836436	0.719613
C	4.644128	-0.622605	-0.775252
S	3.506436	0.664779	-0.987461
H	5.529652	-1.686760	0.921090
H	4.074974	-0.122759	2.519757
H	5.104995	-1.092588	-1.644266
H	2.587462	1.581448	1.100885
C	-4.378544	-1.245858	-1.119466
C	-3.645767	-0.097006	-1.545908
C	-3.171445	0.662450	-0.479007
C	-4.453290	-1.341133	0.270171
S	-3.626762	-0.028223	1.037643
H	-4.833031	-1.978259	-1.790173
H	-3.463699	0.169902	-2.589183
H	-4.946694	-2.105688	0.870609
H	-2.589608	1.583556	-0.497162
C	0.809426	-2.238692	1.339520
C	-0.609824	-2.259098	1.185367
C	-1.010103	-2.016036	-0.127044
C	1.474738	-1.978009	0.142213
S	0.355324	-1.772680	-1.162882
H	1.335567	-2.396914	2.283568
H	-1.321897	-2.428698	1.995810
H	2.546651	-1.894727	-0.037540
H	-2.020862	-1.947756	-0.529193

Cartesian coordinates of : Thio4_30

Atomic number (AN) and Cartesian coordinates

AN	X	Y	Z
C	0.951832	0.848555	-2.769646
C	-0.446127	1.128489	-2.688534
C	-0.810614	1.684204	-1.464010
C	1.635070	1.196583	-1.604548
S	0.560428	1.869811	-0.426312
H	1.446608	0.401573	-3.634989
H	-1.166523	0.926451	-3.484569
H	2.693708	1.078338	-1.371247
H	-1.799882	1.972732	-1.108105
C	-3.964897	0.851368	-0.092508
C	-3.837284	2.173047	0.432143
C	-2.850981	2.259929	1.414731
C	-3.075115	-0.046775	0.492742
S	-2.096380	0.726525	1.688835
H	-4.672004	0.560584	-0.872484
H	-4.433498	3.030079	0.111237
H	-2.936928	-1.107275	0.282159
H	-2.540935	3.134188	1.987590
C	-1.233951	-2.139364	-1.579504
C	-2.050936	-3.152989	-0.993558
C	-1.734689	-3.385646	0.344488
C	-0.304349	-1.612751	-0.687475
S	-0.432269	-2.369055	0.865310
H	-1.319115	-1.788602	-2.609620
H	-2.842716	-3.693923	-1.516703
H	0.439333	-0.833554	-0.853424
H	-2.187301	-4.094925	1.037401
C	3.035993	-1.350591	0.327960
C	4.236887	-0.866567	-0.271388
C	4.620119	0.376847	0.228331
C	2.521542	-0.467997	1.274997
S	3.511441	0.942914	1.432218
H	2.556391	-2.299343	0.079531
H	4.804160	-1.398069	-1.038522
H	1.621637	-0.566745	1.882477
H	5.490561	0.977333	-0.036867

Cartesian coordinates of : Thio4_31

Atomic number (AN) and Cartesian coordinates

AN	X	Y	Z
C	-0.270362	2.211613	1.498552
C	-0.907913	3.335827	0.890594
C	-1.034892	3.189442	-0.490600
C	0.072940	1.224211	0.578093

S	-0.377995	1.679327	-1.028722
H	-0.071561	2.116125	2.568295
H	-1.261155	4.217588	1.429616
H	0.548822	0.260346	0.757588
H	-1.471475	3.885078	-1.207598
C	-3.604801	-0.448125	1.412438
C	-4.284796	-1.536425	0.787670
C	-4.434362	-1.350198	-0.586069
C	-3.241558	0.548093	0.510576
S	-3.741855	0.153269	-1.097227
H	-3.376990	-0.389262	2.478980
H	-4.650583	-2.423686	1.309221
H	-2.705939	1.478760	0.698989
H	-4.914125	-2.007432	-1.311483
C	-0.542304	-2.147956	1.243392
C	0.874821	-2.156411	1.415844
C	1.562353	-1.961379	0.218783
C	-0.919697	-1.951185	-0.082695
S	0.465594	-1.784713	-1.109891
H	-1.270205	-2.263763	2.048632
H	1.384109	-2.288187	2.373190
H	-1.925754	-1.882913	-0.496395
H	2.637705	-1.906647	0.047969
C	4.862981	-0.887239	-0.660879
C	3.981311	-0.157147	-1.513551
C	3.228133	0.787183	-0.819078
C	4.767138	-0.483864	0.670687
S	3.604106	0.781881	0.868060
H	5.540739	-1.675321	-0.996049
H	3.891752	-0.311469	-2.591001
H	5.319363	-0.858474	1.532814
H	2.473761	1.474395	-1.200841

Cartesian coordinates of : Thio4_32

Atomic number (AN) and Cartesian coordinates

AN	X	Y	Z
C	4.245768	0.379922	0.860424
C	4.889749	-0.648864	0.109202
C	4.376615	-0.764221	-1.182036
C	3.253263	1.032032	0.133034
S	3.117240	0.388581	-1.466476
H	4.490012	0.637185	1.893192
H	5.692984	-1.284840	0.487601
H	2.593354	1.840283	0.446301
H	4.677146	-1.451506	-1.973020
C	-0.353823	-2.053759	2.043492
C	1.070863	-1.955634	2.025211
C	1.588346	-1.920302	0.731030
C	-0.905067	-2.091932	0.764600
S	0.325961	-2.018880	-0.449669

H	-0.962698	-2.083115	2.949773
H	1.701851	-1.902056	2.915222
H	-1.954085	-2.152048	0.474350
H	2.627477	-1.834055	0.412537
C	-4.044132	-0.481391	0.774650
C	-4.423802	-1.627094	0.011656
C	-3.843437	-1.634882	-1.256264
C	-3.178508	0.361608	0.082780
S	-2.841232	-0.244196	-1.501188
H	-4.379118	-0.277937	1.793973
H	-5.088458	-2.418034	0.365862
H	-2.714563	1.288767	0.416835
H	-3.957011	-2.375142	-2.048457
C	-0.156247	1.560955	-0.648981
C	-0.000659	1.235490	0.732019
C	-0.506356	2.227380	1.567365
C	-0.783174	2.789781	-0.845137
S	-1.173801	3.549150	0.663485
H	0.161132	0.915342	-1.470114
H	0.451393	0.315428	1.105823
H	-1.034682	3.284310	-1.783616
H	-0.531804	2.248401	2.657033

Cartesian coordinates of : Thio4_33

Atomic number (AN) and Cartesian coordinates

AN	X	Y	Z
C	-0.998205	-2.314428	-1.769016
C	-1.660266	-3.351819	-1.044297
C	-1.386109	-3.302394	0.322034
C	-0.233704	-1.487342	-0.950201
S	-0.323497	-1.988776	0.703484
H	-1.080068	-2.162812	-2.847450
H	-2.311732	-4.105555	-1.491786
H	0.362055	-0.615930	-1.223111
H	-1.748722	-3.961441	1.110957
C	2.868866	-0.477686	1.362959
C	3.181640	-1.233151	0.193162
C	4.290887	-0.730757	-0.481951
C	3.742747	0.589015	1.559859
S	4.947866	0.659284	0.317222
H	2.039681	-0.696888	2.038303
H	2.617359	-2.102117	-0.150786
H	3.744406	1.327323	2.361910
H	4.753897	-1.100366	-1.396972
C	0.947274	1.627560	-2.246434
C	-0.461977	1.836628	-2.144062
C	-0.898482	1.900996	-0.823310
C	1.565488	1.530745	-1.000851
S	0.417803	1.707686	0.284408
H	1.496116	1.540913	-3.186935

H	-1.140050	1.928006	-2.995447
H	2.617149	1.363437	-0.772752
H	-1.910878	2.046734	-0.447693
C	-4.124271	0.570210	-0.650085
C	-4.397628	1.775078	0.065349
C	-3.780711	1.800436	1.315803
C	-3.301110	-0.300699	0.059023
S	-2.874356	0.352861	1.602499
H	-4.501788	0.344397	-1.649657
H	-5.013503	2.595357	-0.309705
H	-2.920144	-1.276634	-0.241067
H	-3.815463	2.584369	2.072504

Cartesian coordinates of :   Thio4_34

Atomic number (AN) and Cartesian coordinates

AN	X	Y	Z
C	-4.360211	1.051641	1.083559
C	-3.663469	-0.186680	1.219538
C	-3.250763	-0.707490	-0.004517
C	-4.465825	1.458646	-0.246360
S	-3.718425	0.319182	-1.313755
H	-4.767993	1.630711	1.914979
H	-3.459765	-0.683410	2.170529
H	-4.941734	2.354180	-0.646168
H	-2.698721	-1.625377	-0.206449
C	-0.539146	2.170996	-1.069754
C	0.884648	2.189296	-1.170341
C	1.512356	1.976839	0.056190
C	-0.982266	1.946714	0.231972
S	0.350816	1.770478	1.324247
H	-1.224727	2.296411	-1.910026
H	1.441123	2.339184	-2.098192
H	-2.005870	1.855817	0.595869
H	2.578656	1.919730	0.275724
C	4.887966	0.872705	0.513087
C	4.121066	-0.012476	1.329067
C	3.323638	-0.873848	0.578411
C	4.661900	0.668540	-0.847877
S	3.518275	-0.601223	-1.116929
H	5.577077	1.629422	0.894282
H	4.143180	-0.024870	2.420869
H	5.110029	1.189909	-1.693982
H	2.632836	-1.641602	0.924988
C	-0.847890	-3.348701	-0.742686
C	-0.325575	-2.166949	-1.351867
C	0.053212	-1.203453	-0.420389
C	-0.852224	-3.268632	0.649680
S	-0.223910	-1.749448	1.197499
H	-1.206216	-4.224116	-1.288644
H	-0.231858	-2.014525	-2.429372

```
 H     -1.187003     -4.016448      1.368956
 H      0.468387     -0.211743     -0.596613
---------------------------------------------------
```

Cartesian coordinates of :   Thio4_35

```
---------------------------------------------------
Atomic number (AN) and Cartesian coordinates
 AN          X             Y             Z
---------------------------------------------------
 C     -3.243710      1.160437      0.194686
 C     -3.214581      0.644521     -1.135644
 C     -4.154046     -0.363380     -1.339350
 C     -4.206510      0.537697      0.983927
 S     -5.073516     -0.672377      0.096677
 H     -2.582543      1.945435      0.566513
 H     -2.534531      0.990077     -1.916605
 H     -4.451514      0.723522      2.029746
 H     -4.356135     -0.931695     -2.247431
 C      3.454093      0.014791     -1.511384
 C      4.102072     -1.256975     -1.499070
 C      4.445671     -1.665900     -0.210584
 C      3.314957      0.558503     -0.236743
 S      3.978836     -0.487501      0.968531
 H      3.092325      0.518890     -2.410062
 H      4.309926     -1.858117     -2.386916
 H      2.858891      1.503346      0.058109
 H      4.945667     -2.583609      0.099419
 C     -0.759118     -2.016095      1.651708
 C      0.636160     -2.096793      1.358111
 C      0.909431     -1.918321      0.004063
 C     -1.532014     -1.768193      0.518652
 S     -0.544730     -1.656013     -0.899863
 H     -1.191390     -2.122702      2.649131
 H      1.420446     -2.262623      2.099477
 H     -2.611646     -1.647310      0.440076
 H      1.875704     -1.908803     -0.500107
 C      1.307722      3.355756      0.914656
 C      0.787337      2.213525      1.596322
 C      0.160673      1.313079      0.738225
 C      1.063150      3.309772     -0.457681
 S      0.205818      1.870567     -0.898206
 H      1.838249      4.179936      1.396404
 H      0.870599      2.043498      2.672019
 H      1.339457      4.039715     -1.218878
 H     -0.313749      0.361079      0.975517
---------------------------------------------------
```

Cartesian coordinates of :   Thio4_36

```
---------------------------------------------------
Atomic number (AN) and Cartesian coordinates
 AN          X             Y             Z
---------------------------------------------------
```

C	-3.477588	-0.470551	1.627836
C	-4.191676	-1.614995	1.160253
C	-4.482261	-1.539746	-0.201720
C	-3.233644	0.458407	0.619893
S	-3.881629	-0.070013	-0.893300
H	-3.143700	-0.326389	2.657589
H	-4.483207	-2.463714	1.782879
H	-2.714625	1.414325	0.683954
H	-5.012635	-2.261527	-0.823119
C	3.254689	1.132013	-0.325604
C	3.259879	0.819302	1.066380
C	4.190347	-0.164585	1.389815
C	4.182026	0.380988	-1.042122
S	5.058099	-0.701421	-0.010815
H	2.593393	1.867535	-0.787797
H	2.607767	1.291947	1.803274
H	4.399403	0.407406	-2.110112
H	4.414075	-0.595517	2.365830
C	-0.462341	-1.465991	-1.549447
C	0.961956	-1.374818	-1.567344
C	1.540250	-1.783302	-0.366383
C	-0.954987	-1.940166	-0.335534
S	0.332146	-2.284409	0.770225
H	-1.116002	-1.185537	-2.378136
H	1.553740	-1.014880	-2.411793
H	-1.990485	-2.087947	-0.029040
H	2.594058	-1.803645	-0.090047
C	-1.003863	3.313211	0.965530
C	-0.296377	2.148872	1.394003
C	-0.008741	1.278300	0.345966
C	-1.237893	3.313753	-0.409469
S	-0.593189	1.890992	-1.160499
H	-1.335418	4.118209	1.624973
H	-0.018042	1.941161	2.429779
H	-1.747293	4.068739	-1.008628
H	0.513467	0.323311	0.368303

Cartesian coordinates of : Thio4_37

Atomic number (AN) and Cartesian coordinates

AN	X	Y	Z
C	-0.083985	1.651652	1.297064
C	-0.711000	2.931368	1.372862
C	-1.045566	3.434535	0.115240
C	0.056518	1.197622	-0.012544
S	-0.578414	2.340360	-1.144148
H	0.234449	1.061269	2.159049
H	-0.921597	3.467903	2.300688
H	0.484617	0.263950	-0.374679
H	-1.525655	4.381478	-0.131498
C	4.154185	-0.278171	1.435959

C	4.921008	-0.943629	0.432657
C	4.664641	-0.448176	-0.845551
C	3.326321	0.709313	0.906046
S	3.492818	0.823584	-0.810133
H	4.198241	-0.507407	2.502839
H	5.630950	-1.750646	0.626259
H	2.627401	1.368607	1.419031
H	5.106366	-0.758805	-1.792614
C	-0.503139	-1.966726	-1.353461
C	0.920160	-1.938622	-1.457539
C	1.550131	-1.979822	-0.214252
C	-0.943359	-2.031131	-0.033014
S	0.391199	-2.065143	1.069640
H	-1.190711	-1.925592	-2.200575
H	1.474493	-1.880037	-2.396866
H	-1.966119	-2.038825	0.343822
H	2.616516	-1.953650	0.010565
C	-4.349364	-1.451151	0.964856
C	-3.662593	-0.319165	1.499104
C	-3.205461	0.547264	0.509111
C	-4.405819	-1.427763	-0.428609
S	-3.622128	-0.022822	-1.067949
H	-4.785293	-2.254122	1.563239
H	-3.500158	-0.138681	2.563971
H	-4.865091	-2.153416	-1.100122
H	-2.650071	1.477926	0.620782

Cartesian coordinates of :   Thio4_38

Atomic number (AN) and Cartesian coordinates

AN	X	Y	Z
C	-4.972117	-1.260459	-0.177856
C	-5.761467	-0.071061	-0.194786
C	-5.006293	1.068376	0.070166
C	-3.631009	-1.011390	0.098278
S	-3.339920	0.678789	0.335648
H	-5.362670	-2.264355	-0.359312
H	-6.835446	-0.040934	-0.392155
H	-2.805827	-1.719039	0.179156
H	-5.342653	2.104406	0.122441
C	-0.520097	-2.806761	0.970271
C	-0.475255	-1.479109	1.492527
C	-0.325099	-0.516169	0.496284
C	-0.409787	-2.830966	-0.422050
S	-0.244120	-1.235171	-1.072247
H	-0.631729	-3.709946	1.573765
H	-0.555163	-1.226888	2.552139
H	-0.419767	-3.696417	-1.084943
H	-0.258459	0.565670	0.598090
C	3.208943	-0.470258	1.425624
C	4.341291	0.290360	1.005018

C	4.796418	-0.087458	-0.257680
C	2.818293	-1.417503	0.483595
S	3.838002	-1.373916	-0.913493
H	2.682966	-0.331309	2.372447
H	4.809080	1.084830	1.591011
H	1.988792	-2.123578	0.532646
H	5.638121	0.311434	-0.824137
C	0.647807	1.903097	-1.601292
C	-0.378472	2.660606	-0.960929
C	-0.016327	3.062439	0.324407
C	1.770648	1.728278	-0.794451
S	1.573880	2.509125	0.735977
H	0.574323	1.490112	-2.609574
H	-1.346630	2.896214	-1.407346
H	2.688552	1.177633	-1.000379
H	-0.593822	3.648797	1.039237
